# Loss of macrophage TSC1 exacerbates sterile inflammatory liver injury through inhibiting the AKT/MST1/NRF2 signaling pathway

**DOI:** 10.1038/s41419-024-06538-4

**Published:** 2024-02-15

**Authors:** Ming Ni, Jiannan Qiu, Guoqing liu, Xiaohu Sun, Wenjie Zhu, Peng Wu, Zheng Chen, Jiajing Qiu, Ziming Wu, Yang Zhang, Feng Zhang, Changyong Li, Yuan Gao, Jun Zhou, Qiang Zhu

**Affiliations:** 1 Hepatobiliary Center, The First Affiliated Hospital of Nanjing Medical University, Nanjing, China; 2 Children’s Hospital of Nanjing Medical University, Nanjing, China; 3 Kangda College of Nanjing Medical University, Lianyun Gang, China; 4 Department of Physiology, Wuhan University School of Basic Medical Sciences, Wuhan, China; 5 Xianning Medical College, Hubei University of Science & Technology, Xianning, China; 6 Department of Hepato-biliary-pancreatic Surgery, The Affiliated Changzhou No.2 People’s Hospital of Nanjing Medical University, Changzhou, China; 7 The Institute of Hepatobiliary and pancreatic diseases, The Affiliated Changzhou No.2 People’s Hospital of Nanjing Medical University, Changzhou, China

**Keywords:** Inflammation, Stress signalling

## Abstract

Tuberous sclerosis complex 1 (TSC1) plays important roles in regulating innate immunity. However, the precise role of TSC1 in macrophages in the regulation of oxidative stress response and hepatic inflammation in liver ischemia/reperfusion injury (I/R) remains unknown. In a mouse model of liver I/R injury, deletion of myeloid-specific TSC1 inhibited AKT and MST1 phosphorylation, and decreased NRF2 accumulation, whereas activated TLR4/NF-κB pathway, leading to increased hepatic inflammation. Adoptive transfer of AKT- or MST1-overexpressing macrophages, or Keap1 disruption in myeloid-specific TSC1-knockout mice promoted NRF2 activation but reduced TLR4 activity and mitigated I/R-induced liver inflammation. Mechanistically, TSC1 in macrophages promoted AKT and MST1 phosphorylation, and protected NRF2 from Keap1-mediated ubiquitination. Furthermore, overexpression AKT or MST1 in TSC1-knockout macrophages upregulated NRF2 expression, downregulated TLR4/NF-κB, resulting in reduced inflammatory factors, ROS and inflammatory cytokine-mediated hepatocyte apoptosis. Strikingly, TSC1 induction in NRF2-deficient macrophages failed to reverse the TLR4/NF-κB activity and production of pro-inflammatory factors. Conclusions: Macrophage TSC1 promoted the activation of the AKT/MST1 signaling pathway, increased NRF2 levels via reducing Keap1-mediated ubiquitination, and modulated oxidative stress-driven inflammatory responses in liver I/R injury. Our findings underscore the critical role of macrophage TSC1 as a novel regulator of innate immunity and imply the therapeutic potential for the treatment of sterile liver inflammation in transplant recipients.

**Figure:**
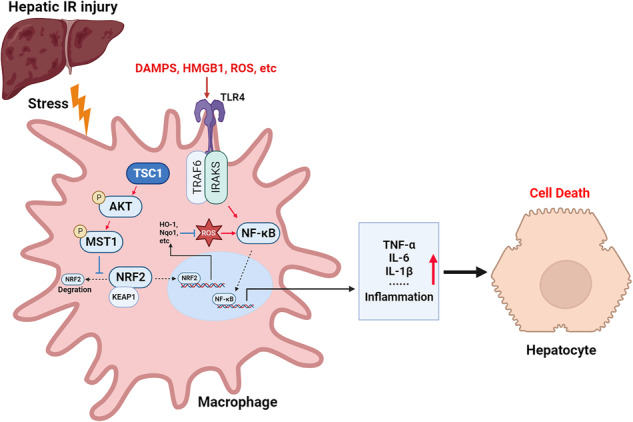
**Schematic illustration of macrophage TSC1-mediated AKT/MST1/NRF2 signaling pathway in I/R-triggered liver inflammation**. Macrophage TSC1 can be activated in I/R-stressed livers. TSC1 activation promotes phosphorylation of AKT and MST1, which in turn increases NRF2 expression and inhibits ROS production and TLR4/NF-κB activation, resulting in reduced hepatocellular apoptosis in I/R-triggered liver injury.

**Schematic illustration of macrophage TSC1-mediated AKT/MST1/NRF2 signaling pathway in I/R-triggered liver inflammation**. Macrophage TSC1 can be activated in I/R-stressed livers. TSC1 activation promotes phosphorylation of AKT and MST1, which in turn increases NRF2 expression and inhibits ROS production and TLR4/NF-κB activation, resulting in reduced hepatocellular apoptosis in I/R-triggered liver injury.

## Introduction

Ischemia/reperfusion (I/R)-induced liver injury is one of the major complications of liver surgery. The excessive inflammatory response caused by activated macrophages exacerbates tissue injury during I/R [1, 2]. Macrophages, as the main components of the innate immune system, are first activated after I/R, and then recruited to hepatic sinuses, directly participating in the liver inflammatory response which is mediated by activating macrophages and adaptive T cells [3–6]. Reactive oxygen species (ROS), and tumor necrosis factor-α (TNF-α) released by activated macrophages promote a sterile inflammatory response, hepatocyte death, and further aggravate I/R-induced liver injury [7–9]. Inhibiting the inflammatory signaling pathway function of activated macrophages can reduce I/R injury [10, 11].

Tuberous sclerosis complex 1 (TSC1) is the upstream molecule of mammalian target of rapamycin (mTOR), which functions by forming a heterodimerc complex with TSC2. TSC2 is a catalytic GTPase-activating protein that can inhibit mTORC1 activity by directly inhibiting Rheb small GTPase upstream of the mTORC1 complex. In hepatocytes, TSC1 can regulate metabolism through the mTOR signaling pathway. Hepatocyte-specific knockout of TSC1 in mice showed the characteristics of decreased activity and hypothermia [12], and reduced liver lipid accumulation induced by a high-fat diet [13]. In a model of high-fat feeding combined with liver I/R injury, hepatocyte-specific knockout of TSC1 attenuated lipid accumulation and liver injury by modulating the mTOR signaling pathway [14]. In macrophages, TSC1 knockout enhanced the sensitivity of macrophages to LPS, promoted M1 macrophage polarization and increased the inflammatory response [15, 16]. Thus, TSC1 is a key regulator for modulating M1/M2 polarization and controlling macrophage inflammatory status to participate in organ tissue damage. However, inhibition of mTOR failed to reverse the M1 type activation or inflammatory response induced by TSC1 deficiency after LPS stimulation [15]. It is possible that there are other downstream molecules involved in TSC1-modulated inflammatory response.

The main function of the Hippo signaling pathway is to inhibit cell growth, and this pathway is composed of a series of kinases, and controls mammalian organ size by regulating cell proliferation, apoptosis and self-renewal [17]. In the I/R-induced acute liver injury model, the Hippo signaling pathway in macrophages plays an important role in regulating the NLRP3-mediated liver inflammation [10]. Mammalian Ste20-like kinase 1/2 (MST1/2) are key components of the Hippo signaling pathway. After activation, MST1/2 promotes the phosphorylation of LATS1/2, which phosphorylates Yap/TAZ, binds with 14-3-3 proteins and finally inhibits the transcriptional coactivation abilty of Yap/TAZ [18, 19]. It has been proven that MST1 plays an important role in regulating the immune system. A previous study showed that MST1 in macrophages can inhibit the inflammatory response by interfering with the Keap1/NRF2 signal axis [20]. Activation of MST1 phosphorylates Keap1, decreases ubiquitination activity and increases the accumulation NRF2, which in turn translocated to the nuclei to activate cytoprotective target genes and participate in the protective effect of liver I/R injury [21–23]. Phosphorylation of AKT, an important downstream molecular protein of PI3K, plays a protective role in I/R-induced liver injury [4, 24]. In addition, a previous report showed that macrophage TSC1 modulated AKT activation [15].

In the present study, we identified a novel functional role and regulatory mechanism of macrophage TSC1 signaling in liver sterile inflammatory injury. We demonstrated that TSC1 in macrophages promoted AKT and MST1 phosphorylation, and then enhanced NRF2 nuclear transcription activity, which was crucial for the modulation of TLR4-driven inflammatory responses. This newly discovered signaling axis provides potential therapeutic targets for the treatment of I/R-triggered liver injury.

## Results

### TSC1 expression is increased in liver macrophages and negatively correlated with I/R-stressed liver injury in patients

**Fig. 1:**
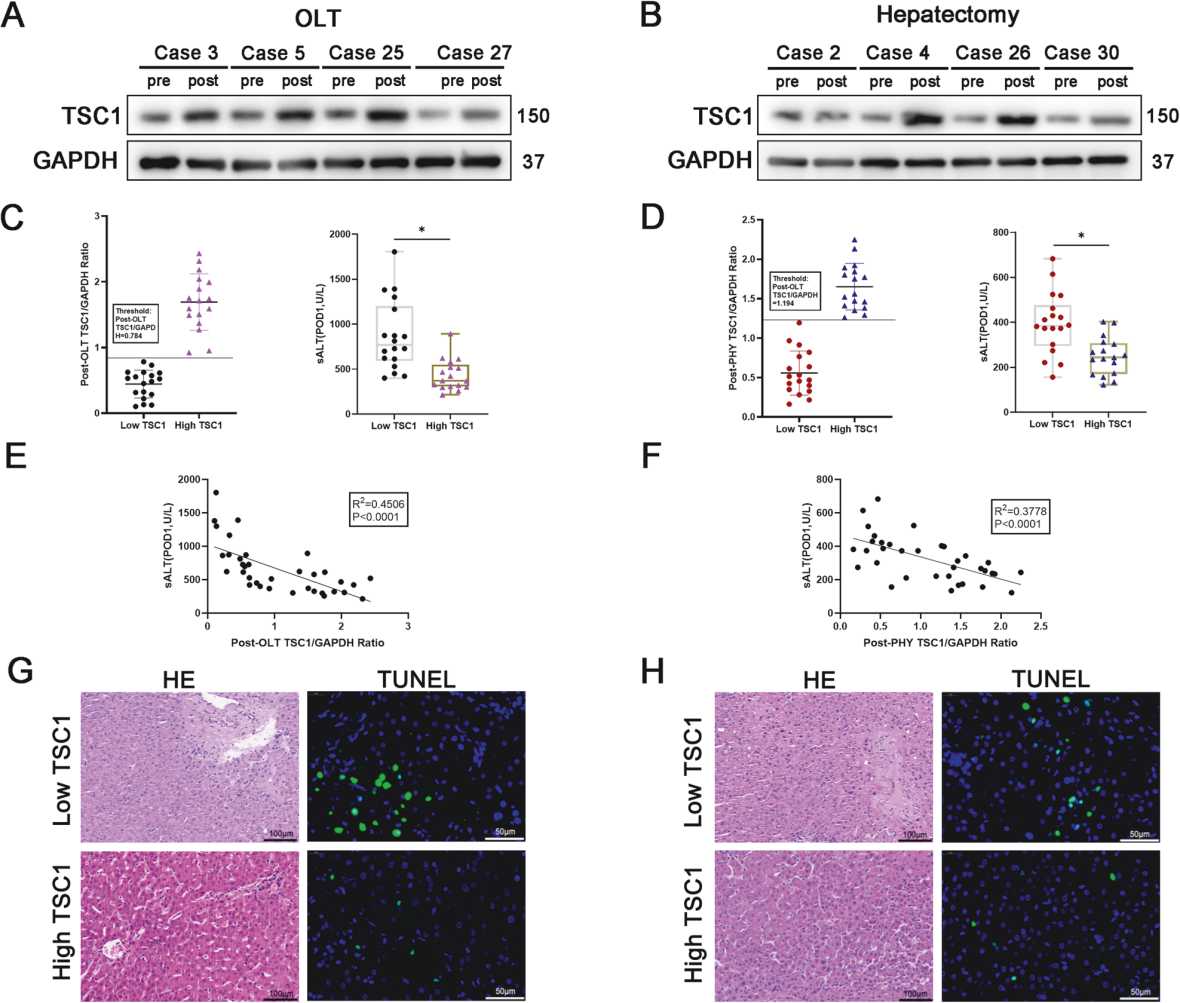
TSC1 expression is increased in liver macrophages and negatively correlated with I/R-stressed liver injury in patients. **A**, **B** Western blot analysis of the relative protein level of TSC1 in macrophage in liver specimens from patients undergoing orthotopic liver transplantation (OLT) or partial hepatectomy (PHY). **C**, **D** Thirty-five human OLTs were divided into low ratio of post-OLT TSC1/GAPDH group (Low TSC1: *n* = 18) and high ratio of post-OLT TSC1/GAPDH group (High TSC1: *n* = 17) by using post-OLT TSC1/GAPDH ratio = 0.784 as threshold. Thirty-five human PHY were divided into low ratio of post-PHY TSC1/GAPDH group (Low TSC1: *n* = 18) and high ratio of post-PHY TSC1/GAPDH group (High TSC1: *n* = 17) by using post-PHY TSC1/GAPDH ratio = 1.194 as threshold. sALT values in both Low TSC1 and High TSC1 groups in OLT recipients or patients undergoing PHY at POD1. **E**, **F** The ratio of post-OLT TSC1/GAPDH and post-PHY TSC1/GAPDH both correlated negatively with sALT at POD1. **G**, **H** Histology (H&E staining) and TUNEL staining of liver specimens from patients undergoing OLT or partial hepatectomy, Scale bars = 100 μm (H&E staining), Scale bars = 50 μm (TUNEL staining). *p < 0.05.

To determine the role of TSC1 in the pathogenesis of liver I/R injury, we first examined TSC1 expression in macrophages in liver specimens from 35 patients undergoing orthotopic liver transplantation (OLT) and 35 patients undergoing partial hepatectomy. The expression of TSC1 was examined in macrophages extracted from fresh liver tissues and further purified with CD68 magnetic beads. Pre-OLT hepatic biopsies were collected during back-table preparation after 2–10 h cold storage (prior to implantation) and post-OLT biopsies were obtained at 3 h after reperfusion (prior to abdominal closure). Pre-hepatectomy hepatic biopsies were harvested after laparotomy (prior to hepatic portal occlusion) and post-hepatectomy hepatic biopsies were obtained at 1.5–2 h after reperfusion (prior to abdominal closure). Ischemic (hepatic portal occlusion) time ranges from 10 to 30 min. The protein level of TSC1 in macrophage from pre/post-OLT liver specimens are shown in (Fig. 1A) (representative of 4 cases) and from pre/post-hepatectomy liver specimens are shown in (Fig. 1B) (representative of 4 cases). The expression of TSC1 in macrophage increased after reperfusion. To evaluate the impact of post-operation TSC1 levels of macrophages for clinical outcomes, we defined these patients into Low TSC1 and High TSC1 group by using post-operation TSC1/GAPDH ratio=0.784 as threshold. Thirty-five human OLTs were divided into low post-OLT TSC1 group (Low TSC1: *n* = 18) and high post-OLT TSC1 group (High TSC1: *n* = 17) (Fig. 1C). Similarly, these 35 patients undergoing partial hepatectomy (PHY) were divided into low post-hepatectomy TSC1 group (Low TSC1: *n* = 18) and high post-hepatectomy TSC1 group (High TSC1: *n* = 17) (Fig. 1D). Unlike Low TSC1 group, patients characterized by higher TSC1 levels exhibited lower sALT at postoperative day 1 (POD1) (Fig. 1C), as did patients who underwent partial hepatectomy (Fig. 1D). Interestingly, the post-operation TSC1 levels correlated negatively with sALT values at POD1 (Fig. 1E: R^2^ = 0.4506, *p* < 0.0001; Fig. 1F: R^2^ = 0.3778, *p* < 0.0001), suggesting that increased TSC1 expression in macrophage was vital for hepatic defend against I/R injury. Moreover, the histology and pathology of specimens in Low TSC1 group from patients undergoing OLT or partial hepatectomy were featured with more severe sinusoidal congestion, edema, vacuolization and apoptosis respectively, as shown by H&E staining and TUNEL staining (Fig. 1G, H).

### Myeloid-specific TSC1 deficiency exacerbates hepatocellular damage in I/R-induced liver injury

**Fig. 2:**
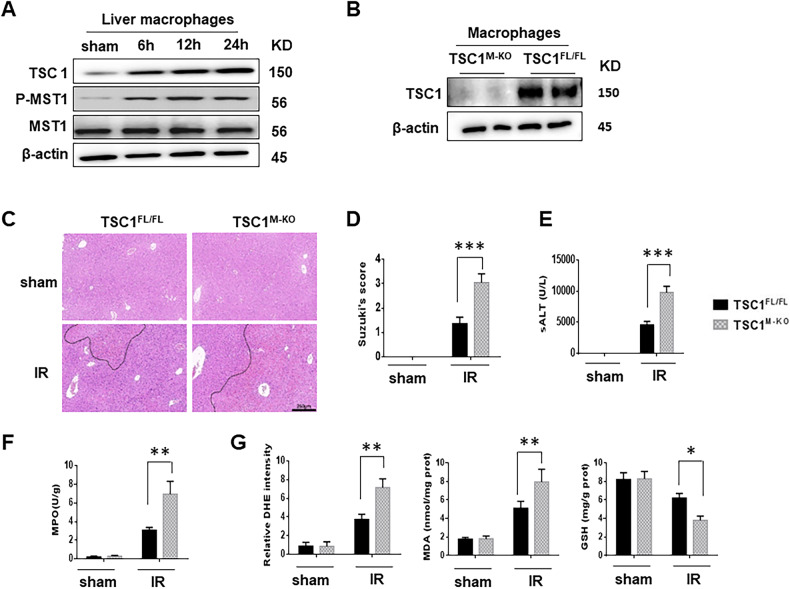
Myeloid-specific TSC1 deficiency exacerbates hepatocellular damage in I/R-induced liver injury. **A** Western-blot analysis of TSC1, p-MST1 and MST1 protein expression in liver macrophages from I/R-stressed livers from mice subjected to 90 min of partial liver warm ischemia, followed by 6 h, 12 h or 24 h of reperfusion. **B** Western-blot analysis of TSC1 in macrophages during I/R. **C** Mice were subjected to 90 min of partial liver warm ischemia, followed by 6 h of reperfusion. Representative histological staining (H&E) of ischemic liver tissue (*n* = 4–6/group), Scale bars = 250 μm. **D** Liver damage, evaluated by Suzuki’s score. ***p < 0.001. **E** Hepatocellular function, as assessed by serum ALT levels (IU/L). The results are expressed as the Mean ± SD (*n* = 4–6 samples/group). ***p < 0.001. **F** Liver neutrophil accumulation, as determined by MPO activity (U/g). The results are expressed as the Mean ± SD (representative of 4–6 mice/group). **p < 0.01. **G** Quantitation of ROS-sensing dye DHE staining, MDA, and GSH activities were assessed in ischemic liver tissues. *p < 0.05, **p < 0.01.

Next, we examined the expressions of TSC1 and MST1 in a mouse model of warm hepatic ischemia followed by reperfusion at various time points [25]. As shown in Fig. 2A, the expressions of TSC1 and MST1 were significantly upregulated in liver macrophages after I/R injury. To determine whether macrophage TSC1 signaling may play a crucial role in liver I/R injury, we generated myeloid-specific TSC1-deficient (TSC1^M-KO^) and TSC1-proficient (TSC1^FL/FL^) mice and subjected them to I/R treatment. We isolated hepatic macrophages and confirmed that TSC1^M-KO^ mice showed TSC1 deficiency in hepatic macrophages compared with TSC1^FL/FL^ mice (Fig. 2B).

Since TSC1 and TSC2 are associated with the mTORC1 signaling pathway [15], we examined the levels of the associated proteins to determine the effect of TSC1 deficiency on the TSC2 and MTORC1 signaling pathway, the result showed that TSC2 was inhibited in the TSC1-deficient bone marrow-derived macrophages (BMMs), as reported that TSC1 stabilized the TSC2 expression [12]. Meanwhile, the protein level of P-p70S6K (T389), P-4E-BP1 (S65) and P-mTOR (S2448) were elevated in TSC1-deficient BMMs, suggesting that the mTORC1 pathway was activated. And the above mTORC1 downstream targets were repressed when the BMMs were pretreated with rapamycin (mTORC1-specific inhibitor) for 24 h (Fig. S1). Compared with the TSC1^FL/FL^ livers, the TSC1^M-KO^ livers showed severe edema, sinusoidal congestion, and necrosis (Fig. 2C, D, score = 3.05 ± 0.36 vs. 1.36 ± 0.27, *p* < 0.001). The levels of serum ALT (IU/L) were significantly higher in the TSC1^M-KO^ mice than that in the TSC1^FL/FL^ controls (Fig. 2E, 9811 ± 1005 vs. 4567 ± 605, *p* < 0.001). The MPO assay, which reflect liver neutrophil activity (U/g), were 3.08 ± 0.3 in the TSC1^FL/FL^ group and 6.96 ± 1.36 in the TSC1^M-KO^ group (Fig. 2F, *p* = 0.0025). In addition, the intracellular ROS production was increased in the TSC1^M-KO^ livers compared with the TSC1^FL/FL^ livers. Consistent with this result, TSC1 deficiency increased the level of dihydroethidium (DHE) and malondialdehyde (MDA) and decreased GSH activity in ischemic livers (Fig. 2G).

### Myeloid-specific TSC1 deficiency promotes hepatocellular apoptosis in I/R-triggered livers

**Fig. 3:**
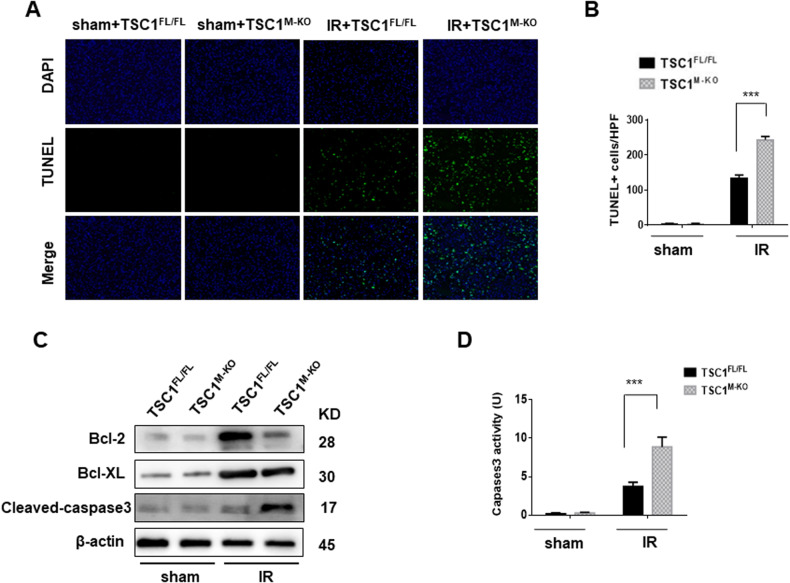
Myeloid-specific TSC1 deficiency increases hepatocellular apoptosis in I/R-triggered livers. **A**, **B** Liver apoptosis analyzed by TUNEL staining, Scale bars = 200 μm. The results were scored semi-quantitatively by averaging the number of apoptotic cells (Mean ± SD) per field at 400× magnification. The results are representative of 4–6 mice/group. ***p < 0.001. **C** Western blot analysis of BCL-2, BCL-xL and Cleaved Caspase 3. β-actin served as an internal control. The data are representative of three experiments. **D** Caspase-3 activity. The results are expressed as the Mean ± SD (*n* = 4–6 samples/group). ***p < 0.001.

To determine the effects of myeloid-specific TSC1 on hepatocellular apoptosis induced by I/R, we performed TUNEL staining to detect apoptotic cells in ischemic livers. TSC1^M-KO^ increased the frequency of TUNEL^+^ cells in the ischemic livers compared to that in the TSC1^FL/FL^ controls (Fig. 3A, B). Consistent with these data, the expressions of anti-apoptotic proteins (Bcl-2 and BCL-xL) were decreased, but the expression of Cleaved Caspase 3 was increased in the TSC1M-KO livers compared with the TSC1FL/FL controls (Fig. 3C). These results were confirmed by increased caspase-3 activity in the TSC1^M-KO^ livers and not in the TSC1^FL/FL^ controls (Fig. 3D).

### Myeloid-specific TSC1 deficiency increases macrophage/neutrophil trafficking, inhibits AKT/MST1/NRF2 activation, and induces TLR4 signaling in I/R-induced liver injury

**Fig. 4:**
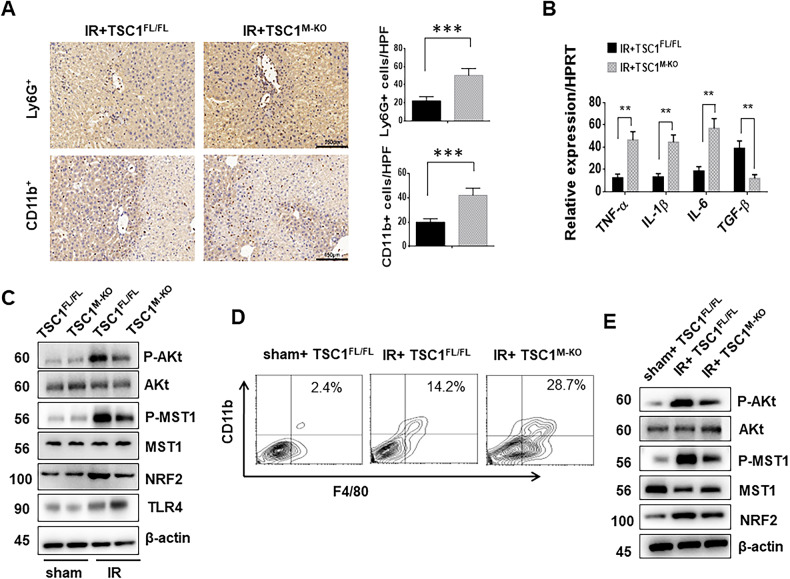
Myeloid-specific TSC1 deficiency increases macrophage/neutrophil trafficking, inhibits AKT/MST1/NRF2 activation, and induces TLR4 signaling in I/R-induced liver injury. **A** Liver CD11b^+^ macrophages and Ly6G^+^ neutrophils were detected by immunohistochemistry. Results were scored semi-quantitatively by averaging the number of positively-stained cells (Mean ± SD)/field at 400× magnification. Representative of 4–6 mice/group, Scale bars = 150 μm. ***p < 0.001. **B** Quantitative RT-PCR-assisted detection of TNF-α, IL-1β, IL-6 and TGF-β expression. Mean ± SD (*n* = 4–6 samples/group). **p < 0.01. **C** Western blot analysis of AKT, phosphorylated AKT, MST1, phosphorylated MST1, NRF2 and TLR4. β-actin served as an internal control. Data are representative of three experiments. **D** Cells were stained with fluorochrome-conjugated anti-F4/80 or -CD11b. F4/80 and CD11b double-positive cells were identified as infiltrating macrophages. **E** Western blot analysis of AKT, p-AKT, MST1, p-MST1 and NRF2 in infiltrating macrophages. β-actin served as an internal control. Data are representative of three experiments.

To determine whether myeloid-specific TSC1 affected the inflammatory cell infiltration in ischemic livers, CD11b^+^ macrophages and Ly6G^+^ neutrophils were detected by immunohistochemistry. CD11b^+^ macrophages and Ly6G^+^ neutrophils were increased in the TSC1^M-KO^ livers compared with the TSC1^FL/FL^ controls (Fig. 4A, 42 ± 5.91 vs. 20 ± 2.91, *p* < 0.001; 50.2 ± 7.66 vs. 22 ± 4.63, *p* < 0.001, respectively). Consistently, TSC1 deletion upregulated TNF-α, IL-1β, and IL-6 and downregulated TGF-β expression in the ischemic livers compared with the TSC1^FL/FL^ controls (Fig. 4B). The protein levels of phospho-AKT, phospho-MST1, and NRF2 were downregulated in parallel with TLR4 upregulation in the TSC1^M-KO^ livers compared with TSC1^FL/FL^ livers (Fig. 4C). In addition, F4/80 and CD11b double-positive macrophages were isolated from normal and I/R livers. The protein expression of phospho-AKT and phospho-MST1 and NRF2 in infiltrating macrophages was higher in the TSC1^FL/FL^ livers than in the TSC1^M-KO^ livers (Fig. 4D, E). These results suggest that myeloid TSC1 plays an important role in the regulation of the innate Hippo and TLR4 signaling pathways during liver inflammatory injury.

### AKT is required for the regulation of MST1/NRF2 in myeloid TSC1-deficient livers in response to I/R stress

**Fig. 5:**
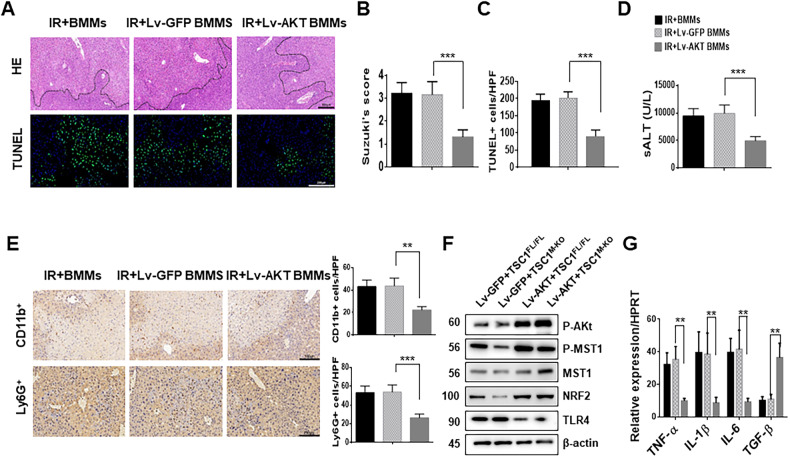
AKT is required for the regulation of MST1/NRF2 in myeloid TSC1-deficient livers in response to I/R stress. Bone marrow-derived macrophages (BMMs) from TSC1^M-KO^ mice were transfected with lentivirus expressing AKT (Lv-AKT) or GFP control (Lv-GFP) and adoptively transferred into TSC1^M-KO^ mice. **A** Representative H&E and TUNEL staining of liver sections (*n* = 4–6 mice/group), Scale bars = 250 μm (H&E), Scale bars = 200 μm (TUNEL). **B** Liver damage, evaluated by Suzuki’s score. ***p < 0.001. **C** The number of apoptotic cells per field at 400× magnification. The results are representative of 4–6 mice/group. ***p < 0.001. **D** The serum ALT levels from the indicated groups. ***p < 0.001. **E** Representative immunohistochemistry staining and quantification of CD11b^+^ macrophages and Ly6G^+^ neutrophils in liver sections (*n* = 4–6 mice/group), Scale bars = 150 μm. **p < 0.01, ***p < 0.001. **F** Western blot analysis of p-AKT, p-MST1, MST1, NRF2, and TLR4. β-actin served as an internal control. Data are representative of three experiments. **G** Quantitative RT-PCR-assisted detection of TNF-α, IL-1β, IL-6 and TGF-β expression. Mean ± SD (*n* = 4–6 samples/group). **p < 0.01.

To determine whether AKT plays a key role in TSC1-mediated immune regulation during liver IRI, AKT activity in ischemic livers was promoted by transfection with lentivirus. We performed transplantation experiments by injecting TSC1-deficient bone marrow-derived macrophages (BMMs) transduced with lentivirus expressing AKT (Lv-AKT) or a GFP control (Lv-GFP) into TSC1^M-KO^ mice. In contrast to the livers treated with Lv-GFP-transfected BMMs or control BMMs, livers collected from the TSC1^M-KO^ mice treated with Lv-AKT-transfected BMMs showed reduced edema, sinusoidal congestion/cytoplasmic vacuolization, and necrosis (Fig. 5A, B, 1.3 ± 0.32 vs. 3.15 ± 0.57, *p* < 0.001), and decreased frequency of TUNEL^+^ cells (Fig. 5A, C). Consistent with the histological data, serum ALT levels (IU/L) were significantly lower in the Lv-AKT-treated TSC1^M-KO^ mice than in the Lv-GFP controls (Fig. 5D, 4951 ± 771 vs. 9934 ± 1485, *p* < 0.001). Moreover, Lv-AKT-transfected cell treatment in TSC1^M-KO^ mice reduced hepatic CD11b^+^ macrophage (Fig. 5E, 22.4 ± 2.96 vs. 43.8 ± 7.04, *p* = 0.0012) and Ly6G^+^ neutrophil recruitment (Fig. 5E, 26.4 ± 3.84 vs. 53.8 ± 7.69, *p* < 0.001) compared with the Lv-GFP-treated controls. The protein expression of phospho-AKT, phospho-MST1 and NRF2 was increased, whereas TLR4 was downregulated (Fig. 5F), which were accompanied by the downregulation of TNF-α, IL-1β, and IL-6 and the upregulation of TGF-β expression in the Lv-AKT-transfected cell-treated livers compared with the Lv-GFP-treated controls (Fig. 5G). These results suggest that AKT is critical for macrophage TSC1-mediated immune regulation during liver I/R injury.

### MST1 overexpression ameliorates myeloid-specific TSC1 deficiency-mediated liver damage in I/R-induced liver injury

**Fig. 6:**
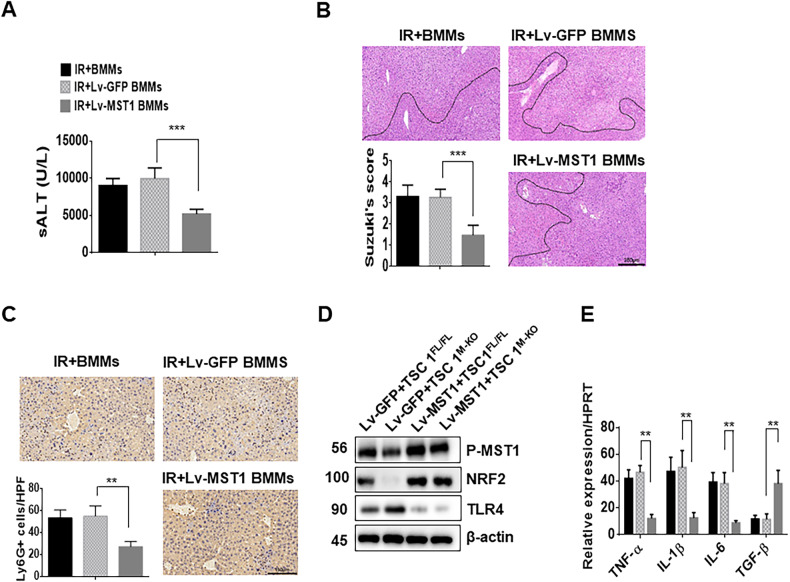
MST1 overexpression ameliorates myeloid-specific TSC1 deficiency-mediated liver damage in I/R-induced liver injury. BMMs from TSC1^M-KO^ mice were transfected with lentivirus expressing MST1 (Lv-MST1) or GFP control (Lv-GFP) and adoptively transferred into TSC1^M-KO^ mice. **A** The serum ALT levels from the indicated groups (*n* = 4–6 mice/group). ***p < 0.001. **B** Representative H&E staining and Suzuki’s score of liver sections (*n* = 4–6 mice/group), Scale bars = 250 μm. ***p < 0.001. **C** Representative immunohistochemistry staining and quantification of Ly6G^+^ neutrophils in liver sections (*n* = 4–6 mice/group), Scale bars = 150 μm. **p < 0.01. **D** Western blot analysis of p-MST1, NRF2, and TLR4. β-actin served as an internal control. Data are representative of three experiments. **E** Quantitative RT-PCR-assisted detection of TNF-α, IL-1β, IL-6 and TGF-β expression. Mean ± SD (*n* = 4–6 samples/group). **p < 0.01.

Next, we evaluated the role of MST1 on TSC1-mediated immune regulation in I/R-stressed livers. Adoptively transferred TSC1-deficient BMMs were transduced with lentivirus expressing MST1 (Lv-MST1) or a GFP control (Lv-GFP) to estimate the function of MST1 overexpression in TSC1^M-KO^ mice. In contrast to livers treated with Lv-GFP-treated controls, livers collected from TSC1^M-KO^ mice treated with Lv-MST1-transfected cells showed significantly improved function (Fig. 6A, 5194 ± 659 vs. 9952 ± 1440, *p* < 0.001), and attenuated edema, sinusoidal congestion/cytoplasmic vacuolization, and necrosis (Fig. 6B, 1.45 ± 0.48 vs. 3.25 ± 0.39, *p* = 0.0002). Moreover, Lv-MST1-transfected cell treatment of the TSC1^M-KO^ mice reduced liver Ly6G^+^ neutrophil recruitment (Fig. 6C, 27.2 ± 4.76 vs. 55.0 ± 9.24, *p* = 0.001) compared with that of the Lv-GFP-treated controls. The protein expression of phospho-MST1 and NRF2 was increased, whereas TLR4 expression was downregulated (Fig. 6D), accompanying by the downregulation of TNF-α, IL-1β, and IL-6, and the upregulation of TGF-β expression in the Lv-MST1-transfected cell-treated livers compared with the levels in the Lv-GFP-treated controls (Fig. 6E). These data demonstrate that MST1 is required for macrophage TSC1-modulated liver inflammatory response.

### ***Silencing of Keap1*** ameliorates TSC1 deficiency-mediated liver damage in I/R-induced liver injury

**Fig. 7:**
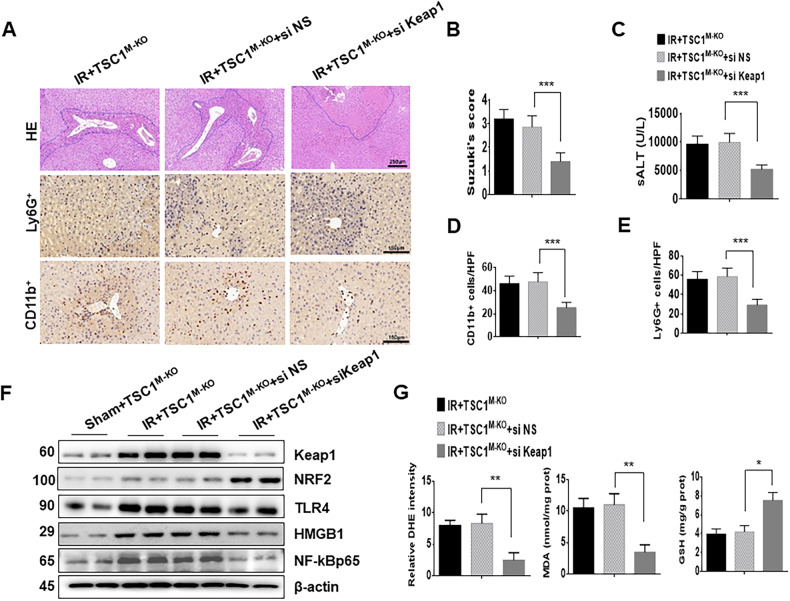
Silencing of Keap1 ameliorates TSC1 deficiency-mediated liver damage in I/R-induced liver injury. TSC1^M-KO^ mice were injected via the tail vein with a mannose-mediated Keap1 siRNA or NS siRNA at 4 h prior to ischemia. **A** Representative histological staining (H&E, original magnification x100) (4–6 mice/group), Scale bars = 250 μm. **B** Liver damage, as evaluated by Suzuki’s score. ***p < 0.001. **C** Hepatocellular function, as assessed by serum ALT levels (IU/L). Results are expressed as the Mean ± SD (*n* = 4–6 samples/group). ***p < 0.001. **D** Liver CD11b^+^ macrophages and (**E**) Ly6G^+^ neutrophils were detected by immunohistochemistry. Results were scored semi-quantitatively by averaging the number of positively-stained cells (Mean ± SD)/field at 400×magnification. Representative of 4–6 mice/group. ***p < 0.001. **F** Western blot analysis of Keap1, NRF2, HMGB1, NF-κB and TLR4. β-actin served as an internal control. Data are representative of three experiments. **G** Quantitation of ROS-sensing dye DHE staining, MDA, and GSH activities were assessed in ischemic liver tissues. *p < 0.05, **p < 0.01.

Next, we evaluated the effect of NRF2 on TSC1-mediated immune regulation in I/R-stressed livers. A Keap1 siRNA with an in vivo mannose-mediated delivery system, which enhances delivery to cells expressing a mannose-specific membrane receptor, was used to transfect macrophages [26]. In contrast to livers treated with NS siRNA, livers collected from TSC1^M-KO^ mice treated with Keap1 siRNA showed decrease dedema, sinusoidal congestion/cytoplasmic vacuolization, and necrosis (Fig. 7A, B, 1.4 ± 0.37 vs. 2.85 ± 0.48, *p* < 0.001), and improved liver function (Fig. 7C, 5251 ± 751 vs. 9995 ± 1565, *p* < 0.001). Consistent with the histological data, Keap1 siRNA treatment in TSC1^M-KO^ mice reduced liver CD11b^+^ macrophage (Fig. 7D, 25.4 ± 4.56 vs. 47.8 ± 7.9, *p* < 0.001) and Ly6G^+^ neutrophil recruitment (Fig. 7E, 29.4 ± 5.83 vs. 58.7 ± 8.7, *p* < 0.001) compared with the NS siRNA controls. We assessed the extent of Keap1 down-regulation after Keap1 siRNA or NS siRNA treatment, which showed that pre-treatment with Keap1 siRNA markedly decreased the protein levels of Keap1 (Fig. 7F). The protein expression of NRF2 was increased, whereas TLR4, HMGB1 and NF-κB were downregulated after Keap1 siRNA treatment (Fig. 7F), which accompanied by the downregulation of ROS production and MDA level, and increased GSH activity (Fig. 7G) in the Keap1 siRNA-treated livers compared with the NS siRNA-treated controls.

### AKT is crucial for TSC1-mediated MST1/NRF2 activation in macrophages in vitro

**Fig. 8:**
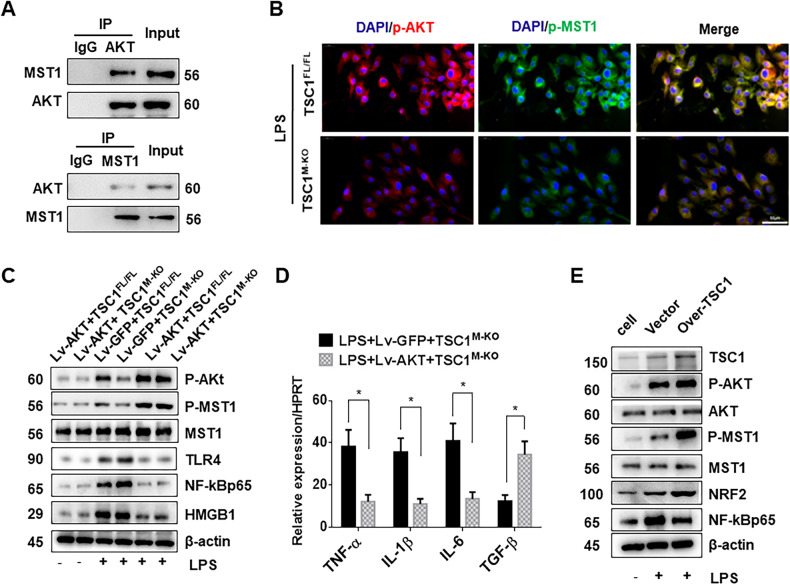
AKT is crucial for TSC1-mediated MST1/NRF2 activation in macrophages in vitro. Bone marrow-derived macrophages (BMMs) were isolated from TSC1^FL/FL^ and TSC1^M-KO^ mice and pretreated with Lv-AKT or Lv-GFP controls before LPS stimulation for 6 h. **A** Immunoprecipitation analysis of AKT and MST1 in macrophages after LPS stimulation. Representative of three experiments. **B** Immunofluorescence localization of DAPI (blue), p-AKT (red) and p-MST1 (green) in LPS-stimulated BMMs, Scale bars = 50 μm. **C** Western blot analysis of phosphorylated AKT, MST1, phosphorylated MST1, HMGB1, NF-κBp65 and TLR4 in LPS-stimulated BMMs. β-actin served as an internal control. **D** Quantitative RT-PCR-assisted detection of TNF-α, IL-1β, IL-6 and TGF-β in LPS-stimulated BMMs. Mean ± SD (*n* = 3–4 samples/group). **p < 0.01. **E** Western blot analysis of TSC1, AKT, phosphorylated AKT, MST1, phosphorylated MST1, NRF2, and NF-κBp65 in LPS-stimulated BMMs followed by Over-TSC1 or Vector-pretreatment. β-actin served as an internal control. Data are representative of three experiments.

To dissect the underlying mechanisms of macrophage TSC1-mediated immune regulation, we performed coimmunoprecipitation assays and immunofluorescent staining in vitro. Coimmunoprecipitation assays revealed that AKT can bind to MST1 in LPS-stimulated BMMs (Fig. 8A). Immunofluorescent staining showed that AKT and MST1 were co-localized in the cytoplasm, and that TSC1 KO inhibited p-AKT and p-MST1 expression (Fig. 8B). This observation was confirmed by Western blots analysis, which showed that TSC1 KO downregulated p-AKT and p-MST1 expression, but upregulated TLR4, NF-κB and HMGB1 in LPS-stimulated BMMs. However, AKT overexpression upregulated phospho-AKT and phospho-MST1, but downregulated TLR4, NF-κB and HMGB1 expression after LPS stimulation (Fig. 8C). Moreover, Lv-AKT treatment decreased TNF-α, IL-1β, and IL-6 expression and increased TGF-β expression compared with the Lv-GFP-treated controls (Fig. 8D). To further determine whether AKT, MST1, NFR2 and NF-κB were specifically modulated by TSC1, we overexpressed TSC1 in BMMs from WT mice. TSC1 overexpression promoted the expression of phospho-AKT and phospho-MST1, upregulated NRF2 and downregulated NF-κB in LPS-stimulated BMMs (Fig. 8E). These results suggest that macrophage TSC1 regulates TLR4 via the AKT/MST1 signaling pathway.

### Macrophage TSC1-mediated MST1 regulates TLR4/NF-κB activity and hepatocyte apoptosis in vitro

**Fig. 9:**
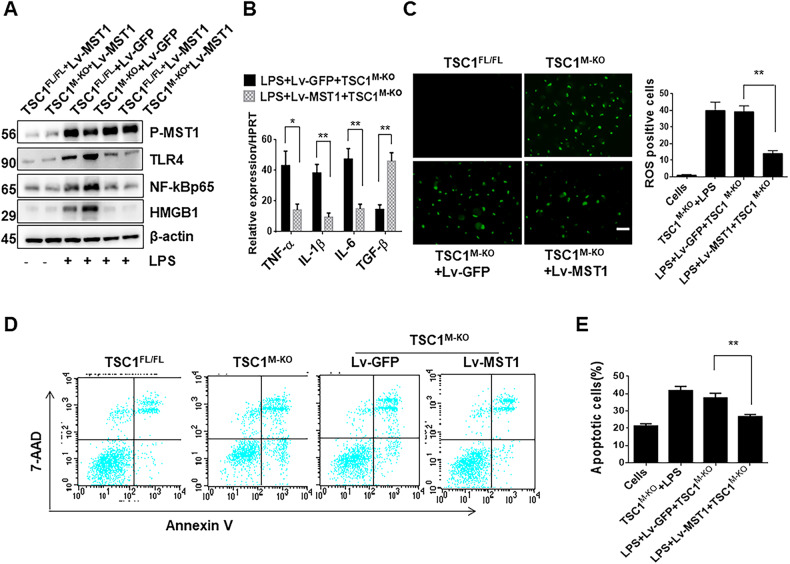
Macrophage TSC1-mediated MST1 regulates TLR4/NF-κB activity and hepatocyte apoptosis in vitro. Bone marrow-derived macrophages (BMMs) were isolated from TSC1^FL/FL^ and TSC1^M-KO^ mice and pretreated with Lv-MST1 or Lv-GFP *controls* before LPS stimulation for 6 h. **A** Western blot analysis of phosphorylated MST1, HMGB1, TLR4, and NF-κBp65 in LPS-stimulated BMMs. β-actin served as an internal control. **B** Quantitative RT-PCR detection of TNF-α, IL-1β, IL-6 and TGF-β in LPS-stimulated BMMs. Mean ± SD (*n* = 3–4 samples/group). **p < 0.01. **C** ROS production was detected by Carboxy-H2DFFDA in LPS-stimulated BMMs from TSC1^M-KO^ mice. Quantification of ROS-producing BMMs (green), Scale bars = 20 μm. **p < 0.01. **D**, **E** Apoptotic hepatocytes from the BMM/hepatocyte coculture system were evaluated by flow cytometry. Representative of three separate experiments. **p < 0.01.

As MST1 plays an important role in regulating the innate immune response, we next investigated the functional role of macrophage MST1 in TSC1-mediated immune regulation. In contrast to the Lv-GFP treated controls, Lv-MST1-mediated overexpression of MST1 in TSC1-deficient BMMs upregulated phospho-MST1 and downregulated HMGB1, TLR4, and NF-κB after LPS stimulation (Fig. 9A). Treatment of Lv-MST1 decreased the mRNA levels of TNF-α, IL-1β, and IL-6, and increased TGF-β expression in response to LPS stimulation compared with Lv-GFP-treated controls (Fig. 9B). These results were accompanied by decreased ROS production (Fig. 9C). Using a BMM/hepatocyte co-culture system, flow cytometry analysis revealed a decreased number of apoptotic hepatocytes after co-culture with Lv-MST1 BMMs compared with that in the Lv-GFP-treated controls (Fig. 9D, E). These results suggest that macrophage MST1 is essential for TSC1-modulating inflammatory responses and hepatocyte apoptosis.

### NRF2 is essential for TSC1-mediated immune regulation in macrophages in vitro

**Fig. 10:**
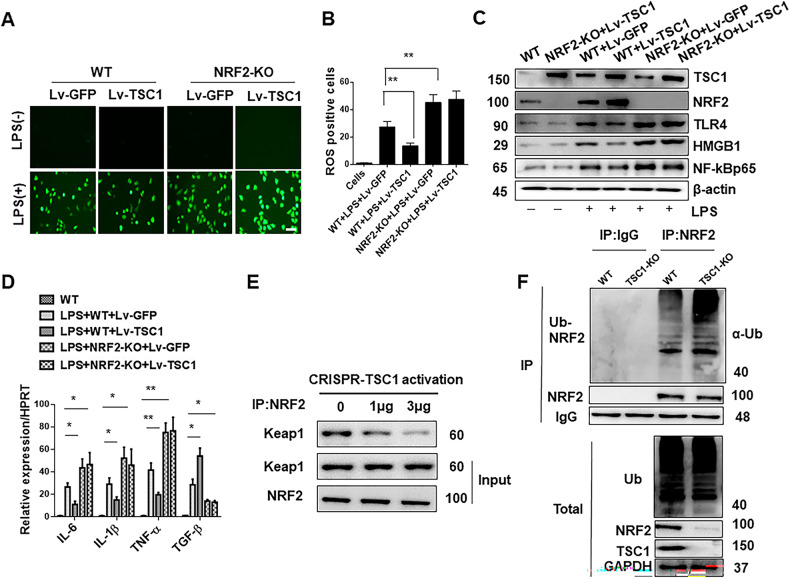
NRF2 is essential for TSC1-mediated immune regulation in macrophages in vitro. Bone marrow-derived macrophages (BMMs) were isolated from NRF2^FL/FL^ and NRF2^M-KO^ mice and pretreated with Lv-TSC1 or Lv-GFP controls before LPS stimulation for 6 h. **A** ROS production was detected by Carboxy-H2DFFDA in LPS-stimulated BMMs from TSC1^M-KO^ mice, Scale bars = 20 μm. **B** Quantification of ROS-producing BMMs (green). **p < 0.01. **C** Western blot analysis of TSC1, NRF2, HMGB1, NF-κB and TLR4. β-actin served as an internal control. Data are representative of three experiments. **D** Quantitative RT-PCR-assisted detection of TNF-α, IL-1β, IL-6 and TGF-β in LPS-stimulated BMMs. Mean ± SD (*n* = 3–4 samples/group). *p < 0.05, **p < 0.01. **E** BMMs were transfected with CRISPR-TSC1 activation vector (1 and 3 µg, respectively). Immunoprecipitation analysis of Keap1 and NRF2 in macrophages after LPS stimulation. **F** Immunoblot analysis of NRF2 ubiquitination in total lysates (bottom) and anti-IgG or anti-NRF2 immunoprecipitates (IP, top) of WT and TSC1-KO BMMs, probed with anti-ubiquitin (α-Ub) and antibodies to NRF2, TSC1, and GAPDH.

As NRF2 plays an important role in regulating the innate immune response, we next investigated the functional role of macrophage NRF2 in TSC1-mediated immune regulation in vitro. In contrast to the Lv-GFP treated controls, Lv-TSC1-mediated overexpression of TSC1 in BMMs decreased ROS production in response to LPS stimulation (Fig. 10A, B). Importantly, NRF2 KO in BMMs increased ROS production compared with WT controls after LPS stimulation. However, TSC1 overexpression in NRF2-deficient BMMs failed to reverse ROS level compared with Lv-GFP-treated controls (Fig. 10A, B). Furthermore, overexpression of TSC1 in BMMs upregulated NRF2 but downregulated HMGB1, TLR4, and NF-κB after LPS stimulation (Fig. 10C). Deletion of NRF2 upregulated HMGB1, TLR4, and NF-κB level, but TSC1 induction in NRF2-deficent BMMs failed to downregulate HMGB1, TLR4, and NF-κB (Fig. 10C). Consistently, treatment of Lv-TSC1 decreased the mRNA levels of TNF-α, IL-1β, and IL-6, and increased TGF-β expression in response to LPS stimulation compared with the levels in Lv-GFP-treated controls. However, overexpression of TSC1 in NRF2-deficient BMMs failed to decrease the mRNA levels of TNF-α, IL-1β, and IL-6, or increase those of TGF-β level (Fig. 10D). Next, we asked how TSC1 affected the expression of NRF2. We found that overexpression of TSC1 significantly reduced the Keap1-NRF2 binding (Fig. 10E). Moreover, NRF2 ubiquitination was increased in TSC1-deficient BMMs but not in WT BMMs (Fig. 10F). These results suggest that NRF2 is essential for TSC1-mediated immune regulation in macrophages.

## Discussion

The present study is the first to demonstrate that TSC1-mediated AKT/MST1/NRF2 signaling is crucial for orchestrating inflammatory responses in sterile liver inflammatory injury. Our data can be summarized as follows: (i) High TSC1 expression was positively correlated with well-preserved histology and improved hepatocellular function in human OLT and hepatectomy. (ii) Myeloid-specific TSC1 deficiency exacerbated I/R-induced liver damage, increased macrophage/neutrophil trafficking, and inhibited AKT and downstream MST1 and NRF2, which in turn activated TLR4/NF-κB; and (iii) AKT is colocalized and interacted with MST1, which in turn regulated NRF2, leading to decreased TLR4/NF-κB activity. These results highlighted the importance of macrophage TSC1-mediated AKT/MST1/NRF2 signaling in the immune regulation during oxidative stress-induced liver inflammation.

TSC1 can reduce the sensitivity of endoplasmic reticulum stress, inhibit the apoptosis induced by endoplasmic reticulum stress and is associated with a variety of immune and inflammatory diseases. Although previous studies showed that TSC1 modulates inflammatory responses by inhibiting TLR4-mediated inflammatory cytokines, and that hepatocyte TSC1 deletion has a protective effect on I/R-induced livers [14], little is known about the exact function and mechanism by which macrophage TSC1 regulates innate TLR4 signaling in I/R-induced liver injury. Our study is the first to document that myeloid-specific TSC1 deletion increased the inflammatory response, as evidenced by the exacerbation of I/R-induced liver damage and increased hepatocellular apoptosis, intrahepatic macrophage/neutrophil accumulation and proinflammatory mediator expression. Moreover, TSC1 deficiency inhibited AKT, MST1 phosphorylation and NRF2 expression and upregulated TLR4/NF-κB in I/R-stressed livers. These results underscored the importance of macrophage TSC1 as a negative regulator of innate TLR4 and its protective role during I/R stress-mediated liver sterile inflammation.

The molecular mechanisms of TSC1-mediated signaling for regulating the innate immune response might involve multiple intercellular pathways. mTOR, as a serine/threonine kinase, is a classical downstream molecule of TSC1, that functions as a core component of two distinct protein complexes, mTORC1 and mTORC2, which regulate different cellular functions [27], namely, cell growth, lipogenesis, protein synthesis, and transcription [28, 29]. TSC1 deficiency promotes M1 macrophage polarization and increases the inflammatory response after LPS stimulation [15, 16]. However, inhibition of mTOR cannot reverse M1-type activation or the TSC1 deficiency-mediated inflammatory response [15]. Thus, TSC1 may participate in the innate immune response via other regulatory mechanisms. To identify the signaling molecules mediating the inflammatory response, we explored specific TSC1 downstream molecules. A previous report showed that macrophage TSC1 disruption inhibited AKT phosphorylation [15]. It is important that AKT, a downstream -+molecule of PI3K, plays a protective role in I/R-induced liver injury [24]. As shown in our results, we found that myeloid specific TSC1 KO inhibited AKT activity, whereas adoptive transfer of AKT-overexpressing BMMs into TSC1^M-KO^ mice reversed TSC1 disruption-induced hepatocellular damage in I/R-triggered liver injury, suggesting that AKT signaling is crucial for TSC1-mediated regulation of the innate TLR4 signaling pathway during liver I/R injury.

Further evidence of TSC1-AKT signaling pathway-mediated modulation of innate TLR4 signaling was obtained from our in vitro study. The results revealed that TSC1 deficiency inhibited AKT phosphorylation and promoted TLR4, NF-κB and HMGB1 in LPS-stimulated BMMs. However, overexpression of AKT in TSC1-deficient BMMs reduced TLR4, NF-κB and HMGB1 expression, which was accompanied by the downregulation of pro-inflammatory cytokines and the upregulation of anti-inflammatory cytokines. Furthermore, the induction of TSC1 promoted AKT activity and downregulated NF-κB in LPS-stimulated BMMs. These results suggested that TSC1-mediated AKT signaling negatively regulated TLR4 activity during the inflammatory response.

Hippo signaling is an evolutionarily conserved pathway that regulates mammalian organ size by controlling cell proliferation, apoptosis and stem cell self-renewal [30]. MST1 is a major member of the Hippo signaling pathway, playing an important role in the innate immune response [31], which is activated in I/R-triggered livers [10]. It has also been reported that the macrophage MST1 signaling pathway can inhibit the inflammatory response by interfering with the Keap1/NRF2 signaling axis [20]. Our previous study also showed that macrophage Hippo signaling pathway plays an important role in I/R-induced liver injury by inhibiting NLRP3-mediated inflammatory response [10]. Consistent with previous study [32], our data also showed that AKT can bind to MST1 in macrophages. Thus, the TSC1-AKT signaling axis may regulate the TLR4-mediated inflammatory response by intervening in MST1/NRF2 activity. In the present study, we found that myeloid-specific TSC1 deletion inhibited AKT and MST1 phosphorylation, leading to decreased NRF2 activity and increased TLR4/NF-κB expression. However, adoptive transfer of MST1-overexpressing BMMs into TSC1^M-KO^ mice reversed TSC1 disruption-induced increase in serum ALT levels, intrahepatic macrophage/neutrophil trafficking, hepatocellular apoptosis, and the upregulation of pro-inflammatory mediators in I/R-triggered liver injury. These results suggest that MST1 mediates the role of TSC1 in the regulation of innate TLR4 during liver I/R injury. Further evidence was obtained from an in vitro study, which showed that overexpression of MST1 in TSC1-KO BMMs downregulated HMGB1 and TLR4/NF-κB, inhibited pro-inflammatory factor levels, and decreased ROS production in LPS-stimulated macrophages. Apoptosis or necrosis is a core mechanism of hepatic function damage in liver IRI, and directly indicates the extent of liver injury. MST1 signaling plays an important role in cytoprotection by increasing antioxidative stress functions [20]. Disruption of NRF2 promoted tissue damage of ischemic-nephrotoxic acute kidney injury in mice, whereas activation of NRF2 decreased the apoptosis/necrosis rates in cerebral I/R tissues [33]. The question arises whether TSC1-mediated MST1/NRF2 signaling regulate hepatocellular apoptosis in I/R-stressed liver. Indeed, our present data showed that TSC1 deficiency decreased the expression of anti-apoptotic BCL-2/BCL-XL, and enhanced caspase-3 activity. Moreover, adoptive transfer of MST1-overexpressing BMMs into TSC1^M-KO^ mice reversed TSC1 disruption-mediated hepatocellular apoptosis in ischemic livers. In addition, in the BMM/hepatocyte co-culture system, induction of MST1 in TSC1-deficient BMMs decreased the number of apoptotic hepatocytes during H_2_O_2_-induced oxidative stress.

In conclusion, we demonstrated that myeloid specific TSC1 deficiency exacerbated I/R-induced liver inflammation by downregulating AKT phosphorylation and the downstream MST1/NRF2 axis, which in turn activated ROS production and TLR4/NF-κB activity, leading to promotion of the sterile liver inflammatory response. By identifying the molecular mechanism by which macrophage TSC1 regulates AKT/MST1/NRF2-mediated innate immunity, our data provide the rationale for novel therapeutic target to attenuate I/R-triggered liver injury.

## Materials and methods

### Patients and specimens

The study was approved by the Research Ethics Committee of the First Affiliated Hospital of Nanjing Medical University, in Nanjing, China (Institutional Review Board approval number 2018-SRFA-197). Biopsy specimens were obtained from 35 patients (Supporting Table 1) with malignant liver disease undergoing orthotopic liver transplantation (OLT). Meanwhile, biopsy specimens were obtained from 35 patients (Supporting Table 2) with benign liver disease undergoing hepatectomy with pringle maneuver. See Supplementary Materials.

### Animals

WT C57BL/6 mice were purchased from the Laboratory Animal Resources of Nanjing Medical University (NMU). Floxed TSC1 (TSC1^FL/FL^) and NRF2 (NRF2^FL/FL^) mice and mice expressing Cre recombinase under the control of the Lysozyme M (LysM) promoter were purchased from GemPharmatech. Co., Ltd. See Supplementary Materials.

### Mouse liver I/R injury model

A mouse model of warm hepatic ischemia followed by reperfusion was used, as previously described [25]. TSC1^M-KO^ mice were injected via the tail vein with TSC1-deficient bone marrow-derived macrophages transfected with lentivirus expressing AKT, MST1 (Lv-AKT, Lv-MST1) or GFP control (Lv-GFP) 24 h prior to ischemia induction, and some TSC1^M-KO^ animals were injected via tail vein with Keap1 siRNA or non-specific (control) siRNA mixed with mannose-conjugated polymers at a ratio according to the manufacturer’s instructions 4 h prior to ischemia as described [26]. See Supplementary Materials.

### Lentiviral vector construction

293 T cells were cotransfected by lentivirus packaging vectors with constructed AKT-, TSC1- or MST1-overexpressing lentivirus. The cells were seeded in six-well plates and transfected when they reached 60–70% confluence. The cells were cotransfected with p-Lv-AKT, p-Lv-TSC1 or p-Lv-MST1, psPAX2 and pVSVG using Lipofectamine 3000 reagent (Invitrogen) to package the lentivirus according to the manufacturer’s instructions. See Supplementary Materials.

### Ubiquitination assay

BMMs were lysed in ice-cold lysis buffer. The cell lysates were subjected to immunoprecipitation with anti-Flag or anti-HA as indicated, were eluted by boiling 10 min in 1% SDS, were diluted ten times in lysis buffer TNTE0.5% and then underwent re-immunoprecipitation with anti-Flag (2×IP). See Supplementary Materials.

### Coculture of macrophages and primary hepatocytes

Primary hepatocytes were cultured in six-well plates at a concentration of 4 × 10^5^ cells per well. After 24 h, 0.4 μm-poresize Transwell inserts (Corning) containing 1 × 10^6^ BMMs were placed into six-well plates with hepatocytes that were initially seeded. The cocultures were incubated for 12 h with or without the addition of H_2_O_2_ (200 µM) to the lower chamber.

### Apoptosis of hepatocytes detection by anexin V/7-AAD flow cytometry

Hepatocytes and their supernatant were centrifuged at 500 × g for 5 min at 4 °C, and then, the supernatant was discarded. After three washes with TBST, the hepatocytes were incubated with annexin V-phycoerythrin and 7-amino-actinomycin D (7-AAD) according to the manufacturer’s directions. See Supplementary Materials.

### Reactive oxygen species assay

ROS production in BMMs was measured using a carboxy-H2DFFDA kit as described [34]. The ROS produced by the BMMs was analyzed and quantified by fluorescence microscopy. Positive green fluorescent-labeled cells were counted blindly at 10 HPF/section (200×). See Supplementary Materials.

### Hepatocellular function assay

Serum alanine aminotransferase (sALT) levels, an indicator of hepatocellular injury, were measured by an automated chemical analyzer. See Supplementary Materials.

### Histology

Liver sections were stained with hematoxylin and eosin (H&E). The severity of the IRI was graded using Suzuki’s criteria on a scale from 0 to 4 [35]. Superoxide levels in liver tissues were assessed by ROS-sensing dye dihydroethdium. Liver sections were evaluated blindly by counting labeled cells in 10 HPF. See Supplementary Materials.

### Immunohistochemistry staining

Liver macrophages and neutrophils were detected using primary rat anti-mouse CD11b^+^ mAb or Ly6G^+^ mAb. After incubation with secondary biotinylated goat anti-rat IgG, followed by treatment with immunoperoxidase, positive cells were counted blindly at 10 HPF/section (x400). The samples were premounted with VECTASHIELD medium with DAPI, p-AKT and p-MST1 in macrophages were detected using mouse anti-mouse p-AKT or rabbit anti-mouse p-MST1 Ab as a primary antibody, and macrophages were incubated with immunoperoxidase according to the manufacturer’s instructions. See Supplementary Materials.

### Immunoprecipitation analysis

BMMs after LPS stimulation were lysed in NP-40 lysis buffer. The lysates were incubated overnight with AKT, MST1 antibody or control IgG and protein A/G beads at 4 °C. After immunoprecipitation, the immunocomplexes were analyzed by standard immunoblot procedures. See Supplementary Materials.

### Myeloperoxidase activity assay

The presence of myeloperoxidase (MPO) was used as an index of hepatic neutrophil accumulation [2]. See Supplementary Materials.

### Malondialdehyde (MDA) and Glutathione (GSH)

MDA and GSH activities were assessed in ischemic liver tissues 6 h after reperfusion. The activities were measured using MDA and GSH assay kits (Jiancheng Biotechnology) according to the manufacturer’s instructions.

### TUNEL staining

Liver sections (4 μm) were stained via terminal deoxynucleotidyl transferase dUTP nick end labeling (TUNEL) using an in situ cell death detection kit (Roche-Boehringer Mannheim, Germany) according to the manufacturer’s instructions as previously described [2]. See Supplementary Materials.

### Caspase-3 activity assay

Caspase-3 activity was determined by an assay kit (Calbiochem, La Jolla, CA), as previously described [2]. See Supplementary Materials.

### Quantitative RT-PCR analysis

Quantitative real-time PCR was performed using the DNA Engine with Chromo 4 Detector (MJ Research, Waltham, MA). In a final reaction volume of 25 μl, the following were added: 1×SuperMix (Platinum SYBR Green qPCR Kit; Invitrogen, San Diego, CA) cDNA and 10 μM of each primer. Amplification conditions were: 50 °C (2 min), 95 °C (5 min), followed by 40 cycles of 95 °C (15 s) and 60 °C (30 s). Primer sequences used for the amplification were shown in Supporting Table 3.

### Western blot analysis

Protein was extracted from liver tissue or cell cultures, as described [22]. Details and antibodies used in our study are present in Supplementary Materials.

### BMM isolation and in vitro transfection

Murine bone marrow-derived macrophages (BMMs) were generated as previously described [36]. In brief, BMMs were transfected with Lv-TSC1, Lv-MST1, Lv-AKT activation or Lv-GFP control vector. Overexpression of TSC1 was established as described [37], BMMs were transfected with one of the following constructs: tuberous sclerosis complex 1 (TSC1, 500 ng), or EGFP vector (500 ng) per well using Lipofectamine reagent according to the manufacturer’s instructions for 48 h. BMMs (1 × 10^6^/well) transfected with CRISPR-TSC1 activation, or control vector (Santa Cruz Biotechnology). After 24–48 h, cells were supplemented with 100 ng/ml of LPS for additional 6 h. Rapamycin (RAPA, 20 nM) was used to treat BMMs for 24 h. See Supplementary Materials.

### Flow cytometry analysis

Liver NPCs were isolated from sham or I/R livers, as described above [38]. A total of 1 × 10^6^ cells were incubated with purified rat anti-mouse CD16/32 for 10 min and stained with rat anti-mouse F4/80-PE, CD11b-FITC and isotype-matched negative control Abs were added to the cell suspension. After 20 min of incubation in the dark, the cells were washed with PBS and subjected to flow cytometric analysis with FACS Calibur (BD Biosciences). See Supplementary Materials.

### Statistical analysis

Data are expressed as Mean ± SD and analyzed by Student’s *t* tests. Per comparison two-sided *p* values less than 0.05 were considered statistically significant. Multiple group comparisons were performed using one-way ANOVA with a post-hoc test. All statistical analysis was performed using SPSS software.

### Supplementary information

Supplemantary Figure and tables

Supplementary Materials

Original Data File

aj-checklist

## Data Availability

The data during the current study are available from the corresponding author on a reasonable request.
