# Supplementary material for: Loss of macrophage TSC1 exacerbates sterile inflammatory liver injury through inhibiting the AKT/MST1/NRF2 signaling pathway
Source: Cell Death Dis. 2024 Feb 15;15(2):146. doi: 10.1038/s41419-024-06538-4 (PMC10869801; doi:10.1038/s41419-024-06538-4)
Supplement: Supplementary file 1 — Supplemantary Figure and tables [file 41419_2024_6538_MOESM1_ESM.pdf]

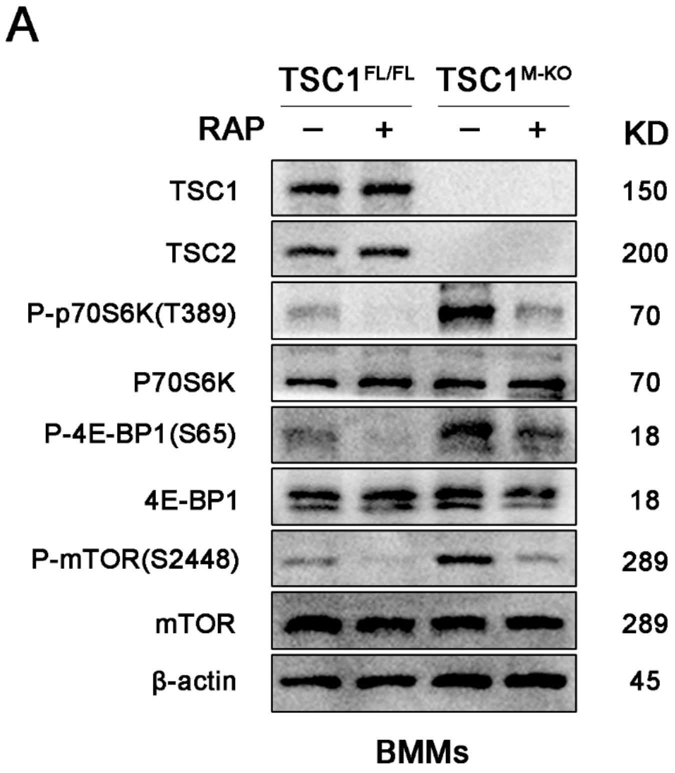

FigS1 TSC1 deficiency reduced the expression of TSC2 and activates the mTORC1 signaling pathway

A. Western blot analysis of TSC1, TSC2, P-p70S6K (T389), p70S6K, P-4E-BP1 (S65), 4E-BP1, P-mTOR (S2448) and mTOR. β-actin served as an internal control. RAPA (Rapamycin, 20nM) was used to treat BMMs for 24h. The data are representative of three experiments.

Supporting Table 1 OLT Patient characteristics

VARIABLES	PATIENTS
N	35
SEX (MALE/FEMALE)	21/14
AGE (YEAR)	55.62± 7.39
AETIONLOGY OF DISEASE (N)	NORMAL LIVER(35)
ISCHEMIA TIME(H)	2-10
REPERFUTION TIME(H)	2-3
ALT OF POD1(U/L)	915.748±888.381

Supporting Table 2 PHY Patient characteristics

VARIABLES	PATIENTS
N	35
SEX (MALE/FEMALE)	20/15
AGE (YEAR)	49.56 ± 9.41
AETIONLOGY OF DISEASE (N)	HEPATIC HEMANGIOMA (24)<br>INTRAHEPATIC CHOLELITHIASIS(5)<br>HEPATIC CYST (4)<br>FNH (2)
ISCHEMIA TIME(MIN)	15-30
REPERFUTION TIME(H)	1.5-2
ALT OF POD1(U/L)	402.4±280.27

Supplementary Table 3: Primer sequences for the amplification

Target genes	Forward primers	Reverse primers
HPRT	5’-TCAACGGGGGACATAAAAGT-3’	5’-TGCATTGTTTTACCAGTGTCAA-3’
TNF-α	5’-ACGGCATGGATCTCAAAGAC-3’	5’-AGATAGCAAATCGGCTGACG-3’
Il-6	5’-CTCTGGGAAATCGTGGAAATG-3’	5’-AAGTGCATCATCGTTGTTCATACA-3’
IL-1β	5’-GCGGCCAGGATATAACTGACTTC -3’	5’-GCGGCCAGGATATAACTGACTTC-3’
TGF-β	5’-TGCGCTTGCAGAGATTAAAA -3’	5’-CTGCCGTACAACTCCAGT -3’
