# Supplementary material for: Loss of macrophage TSC1 exacerbates sterile inflammatory liver injury through inhibiting the AKT/MST1/NRF2 signaling pathway
Source: Cell Death Dis. 2024 Feb 15;15(2):146. doi: 10.1038/s41419-024-06538-4 (PMC10869801; doi:10.1038/s41419-024-06538-4)
Supplement: Supplementary file 2 — Supplementary Materials [file 41419_2024_6538_MOESM2_ESM.docx]

**Supplementary Materials**

***Patients and specimens***

The study was approved by the Research Ethics Committee of the First Affiliated Hospital of Nanjing Medical University, in Nanjing, China (Institutional Review Board approval number 2018-SRFA-197). Biopsy specimens were obtained from 35 patients (Supporting Table 1) with malignant liver disease undergoing orthotopic liver transplantation (OLT) (January 2019-June 2020, Hepatobiliary Center, the First Affiliated Hospital of Nanjing Medical University). Pre-OLT hepatic biopsies were harvested from the left lobe during back-table preparation (prior to implantation), and post-OLT hepatic biopsies were harvested at 2-3h after portal reperfusion (prior to the abdominal closure). All organ donations recorded in the electronic database were contributed voluntarily, and no donor livers were obtained from executed prisoners or other institutionalized persons. Non-fatty donor organs, procured according to standardized techniques, were perfused with and stored in cold University of Wisconsin (UW) solution. The ischemia time was defined as the time between the perfusion of the donor liver with UW solution and its removal from the cold storage. Meanwhile, biopsy specimens were obtained from 35 patients (Supporting Table 2) with benign liver disease undergoing hepatectomy with pringle maneuver (January 2019-June2020, Hepatobiliary Center, the First Affiliated Hospital of Nanjing Medical University). Pre-hepatectomy hepatic biopsies were harvested after laparotomy (prior to hepatic portal occlusion) and post-hepatectomy hepatic biopsies were obtained after reperfusion (prior to abdominal closure). Ischemic time is from 15 to 30 minutes (min). Serum alanine aminotransferase (sALT) was detected on the first day postoperatively (POD1) to assess the degree of hepatocellular injury. Informed consent was obtained from all participants.

***Animals.***

WT C57BL/6 (male, 6-8 week old) mice were purchased from the Laboratory Animal Resources of Nanjing Medical University (NMU). Floxed TSC1 (TSC1^FL/FL^) and NRF2 (NRF2^FL/FL^) mice and mice expressing Cre recombinase under the control of the Lysozyme M (LysM) promoter were purchased from GemPharmatech. Co., Ltd. Eight-week-old TSC1^FL/FL^, TSC1^M-KO^, NRF2 ^FL/FL^ and NRF2 ^M-KO^ male mice were used in all experiments. The mice used in this study had the same gender, almost equal weight and randomized (simple randomization) into groups. This study was performed in strict accordance with the recommendations in the *Guide for the Care and Use of Laboratory Animals* published by the National Institutes of Health. The animal protocol was approved by the Institutional Animal Care & Use Committee (IACUC) of Nanjing Medical University.

***Mouse liver IRI model.***

A mouse model of warm hepatic ischemia followed by reperfusion was used, as previously described [25]. Mice were injected with heparin (100U/kg) and an atraumatic clip was used to interrupt the arterial/portal venous blood supply to the cephalad liver lobes. After 90min the clip was removed, and mice were sacrificed at 6h of reperfusion. TSC1^M-KO^ mice were injected via the tail vein with TSC1-deficient bone marrow-derived macrophages (BMMs, 5×10^6^ cells/mouse) transfected with lentivirus expressing AKT, MST1 (Lv-AKT, Lv-MST1) or GFP control (Lv-GFP) 24h prior to ischemia induction, and some TSC1^M-KO^ animals were injected via tail vein with Keap1 siRNA or non-specific (control) siRNA, (2mg/kg) (Santa Cruz Biotechnology, Shanghai, China) mixed with mannose-conjugated polymers (Polyplus transfection™, Illkirch, France) at a ratio according to the manufacturer’s instructions 4h prior to ischemia as described [26].

***Lentiviral vector construction.***

293T cells were cotransfected by lentivirus packaging vectors with constructed AKT-, TSC1- or MST1-overexpressing lentivirus. The cells were seeded in six-well plates and transfected when they reached 60–70% confluence. The cells were cotransfected with p-Lv-AKT, p-Lv-TSC1 or p-Lv-MST1, psPAX2 and pVSVG using Lipofectamine 3000 reagent (Invitrogen) to package the lentiviruses according to the manufacturer’s instructions. The following amounts of plasmid DNA were used per well: 1 μg of p-Lv-AKT, p-Lv-TSC1 or p-Lv-MST1 (or control vector), 0.625μg of psPAX2, and 0.375μg of pCMV-VSVG. Forty-eight hours after transfection, the viral vector-containing supernatant was collected and filtered through a 0.45 μm filter. The AKT, TSC1 or MST1 lentivirus (Lv-AKT, Lv-TSC1 or Lv-MST1) was either immediately used or snap-frozen at −80℃ for later use. GFP lentivirus (Lv-GFP, Applied Biological Materials Inc.) was used as a control. 293T cells and identification are provided by the cellular company, and mycoplasma contamination is regularly tested in our laboratory.

***Ubiquitination assay.***

BMMs were lysed in ice-cold lysis buffer (“TNTE 0.5%”: 50 mM Tris-HCl, pH7.5, 150 mM NaCl, 1 mM EDTA and 0.5% Triton X-100, containing 10 mM NaF, 2 mM Na3VO4, 10 mg ml^−1^ leupeptin and 1 mM PMSF). The cell lysates were subjected to immunoprecipitation with anti-Flag or anti-HA as indicated, were eluted by boiling 10 min in 1% SDS, were diluted ten times in lysis buffer TNTE0.5% and then underwent re-immunoprecipitation with anti-Flag (2×IP). The ubiquitin-conjugated proteins were detected by immunoblot analysis with the appropriate antibodies

***Coculture of macrophages and primary hepatocytes.***

Primary hepatocytes were cultured in six-well plates at a concentration of 4×10^5^ cells per well. After 24h, 0.4μm-poresize Transwell inserts (Corning) containing 1×10^6^ BMMs were placed into six-well plates with hepatocytes that were initially seeded. The cocultures were incubated for 12h with or without the addition of H_2_O_2_ (200µM) to the lower chamber.

***Apoptosis of hepatocytes detection by anexin V/7-AAD flow cytometry.***

Hepatocytes and their supernatant were centrifuged at 500× g for 5min at 4℃, and then, the supernatant was discarded. After three washes with TBST, the hepatocytes were incubated with annexin V-phycoerythrin and 7-amino-actinomycin D (7-AAD) according to the manufacturer’s directions (apoptosis detection Kit; BD Biosciences). The percentage of apoptotic cells was determined by flow cytometry (BD Biosciences).

***Reactive oxygen species assay***

ROS production in BMMs was measured using a carboxy-H2DFFDA kit as described [34]. In brief, BMMs from NRF2^FL/FL^, TSC1^FL/FL^, NRF2^M-KO^ and TSC1^M-KO^ mice were transfected with Lv-TSC1, Lv-MST1 activation or Lv-GFP control vector respectively and then cultured on collagen-coated cover slips after LPS stimulation. After washing with PBS, the cells were incubated with 10 μM carboxy-H2DFFDA. Carboxy-H2DFFDA was converted to a fluorescent green form when it was hydrolyzed by intracellular esterases and oxidized in cells. The cells were then fixed with 2% paraformaldehyde and stained with Hoechst dye. The ROS produced by the BMMs was analyzed and quantified by fluorescence microscopy. Positive green fluorescent-labeled cells were counted blindly at 10 HPF/section (200×).

***Hepatocellular function assay.***

Serum alanine aminotransferase (sALT) levels, an indicator of hepatocellular injury, were measured by an automated chemical analyzer (Olympus Automated Chemistry Analyzer AU5400, Tokyo, Japan).

***Histology.***

Liver sections were stained with hematoxylin and eosin (H&E). The severity of the IRI was graded using Suzuki’s criteria on a scale from 0 to 4 [35]. In this classification, no necrosis, congestion or centrilobular ballooning was given a score of 0, while severe congestion and ballooning degeneration and >60% lobular necrosis were given a value of 4. Superoxide levels in liver tissues were assessed by ROS-sensing dye dihydroethdium (DHE; Jiangsu Aoyin Medical Technology Co., Ltd, Nanjing, China). Liver sections were evaluated blindly by counting labeled cells in 10 high-power fields (HPF).

***Immunohistochemistry staining.***

Liver macrophages and neutrophils were detected using primary rat anti-mouse CD11b^+^ mAb (Mac-1, M1/70; BD Biosciences, San Jose, CA) or Ly6G^+^ mAb (BD Biosciences, San Diego, CA). After incubation with secondary biotinylated goat anti-rat IgG (Vector, Burlingame, CA), followed by treatment with immunoperoxidase (ABC Kit, Vector), positive cells were counted blindly at 10 HPF/section (x400). The samples were premounted with VECTASHIELD medium with DAPI. p-AKT and p-MST1 in macrophages were detected using p-AKT Ab or p-MST1 Ab as a primary antibody, and macrophages were incubated with immunoperoxidase (ABC Kit, Vector) according to the manufacturer’s instructions. The positive cells were counted blindly at 10 HPF/section (200×).

***Immunoprecipitation analysis.***

BMMs after LPS stimulation were lysed in NP-40 lysis buffer. The lysates were incubated overnight with Akt (CST #4691), MST1 (ABCAMab51134) antibody or control IgG and protein A/G beads at 4℃. After immunoprecipitation, the immunocomplexes were analyzed by standard immunoblot procedures.

***Myeloperoxidase activity assay.***

The presence of myeloperoxidase (MPO) was used as an index of hepatic neutrophil accumulation [2]. The change in absorbance was measured spectrophoto metrically at 655 nm. One unit of MPO activity was defined as the quantity of enzyme that degrades 1μmol peroxide/min at 25℃ per gram of tissue.

***Malondialdehyde (MDA) and Glutathione (GSH)***

MDA and GSH activities were assessed in ischemic liver tissues 6h after reperfusion. The activities were measured using MDA and GSH assay kits (Jiancheng Biotechnology) according to the manufacturer’s instructions.

***TUNEL staining.***

Liver sections (4μm) were stained via terminal deoxynucleotidyl transferase dUTP nick end labeling (TUNEL) using an in situ cell death detection kit (Roche-Boehringer Mannheim, Germany) according to the manufacturer’s instructions as previously described [2].The apoptosis rate of hepatocytes was measured by using a TUNEL Apoptosis Assay Kit (Roche-Boehringer Mannheim, Germany). DAPI (Sigma) was used to counterstain the nuclei and the TUNEL-positive cells were excited with CY3 at a wavelength of 510-561nm, the emission wavelength of was 590nm, and green light emission was visually identified by fluorescence microscopy using a FITC filter. The results were scored semiquantitatively by averaging the number of apoptotic cells/microscopic field at 200× magnification. Ten fields were evaluated per sample.

***Caspase-3 activity assay.***

Caspase-3 activity was determined by an assay kit (Calbiochem, La Jolla, CA), as previously described [2]. Liver tissues were collected and resuspended in lysis buffer containing 50 mmol/L HEPES, pH7.4, 0.1% CHAPS, 1 mmol/L DTT, 0.1mmol/L EDTA and 0.1% Triton X-100. Following incubation for 30min on ice, cell lysate was centrifuged at 16000g for 10 min at 4℃，and the protein concentration in the supernatants was measured using the Bradford dye method. The supernatants were incubated with 200μM of enzyme-specific colorimetric caspase-3 substrate at 37℃for 2h. Caspase-3 activity was assessed by measuring the absorbance at a wavelength of 405nm with a plate reader. To determine cellular activity, the inhibitor-treated protein extracts and the purified caspase-3 (as a standard) were used.

***Quantitative RT-PCR analysis.***

Quantitative real-time PCR was performed using the DNA Engine with Chromo 4 Detector (MJ Research, Waltham, MA). In a final reaction volume of 25μl, the following were added: 1× SuperMix (Platinum SYBR Green qPCR Kit; Invitrogen, San Diego, CA) cDNA and 10μM of each primer. Amplification conditions were: 50℃ (2min), 95℃ (5min), followed by 40 cycles of 95℃ (15sec) and 60℃ (30sec). Primer sequences used for the amplification were shown in Supplementary Table 3.

***Western blot analysis.***

Protein was extracted from liver tissue or cell cultures, as described [22]. Protein samples were separated by 10% SDS-PAGE and transferred onto polyvinylidene fluoride (PVDF) membranes. After blocking the PVDF membranes in QuickBlock™ Blocking Buffer (Beyotime Biotechnology) for at least 30min, we incubated them with specific primary antibodies at 4℃ for 12 h. After washing the PVDF membranes with Tris-buffered saline (TBST) buffer for 30min, we incubated them with horseradish peroxidase (HRP) -conjugated anti-rabbit IgG antibodies at room temperature for 2h. Finally, the proteins were detected using the Super ECL Detection Reagent (Yeasen Biotech Co., Ltd) after rinsing with TBST buffer for 30min. The relative quantities of proteins were determined by a densitometer, and the results are expressed in absorbance units (AU).

***BMM isolation and in vitro transfection.***

Murine bone marrow-derived macrophages (BMMs) were generated as previously described [36]. In brief, bone marrow cells were removed from the femurs and tibias of TSC1^FL/FL^, NRF2^FL/FL^, NRF2^M-KO^ and TSC1^M-KO^ mice and cultured in DMEM supplemented with 10% FCS and 20% L929-conditioned medium. Cells (1x10^6^/well) were cultured for 7 days and then transfected with Lv-TSC1, Lv-MST1, Lv-AKT activation or Lv-GFP control vector. After 24-48h, cells were supplemented with 100ng/ml of LPS for additional 6h. Overexpression of TSC1 was established as described [37], BMMs were transfected with one of the following constructs: tuberous sclerosis complex 1 (TSC1, 500ng), or EGFP vector (500ng) per well using Lipofectamine reagent according to the manufacturer’s instructions for 48h. BMMs (1×10^6^/well) transfected with CRISPR-TSC1 activation, or control vector (Santa Cruz Biotechnology). After 24–48h, cells were supplemented with 100ng/ml of LPS for additional 6h. To evaluate the effect of TSC1 deficiency on the expression of TSC2 and mTOR，BMMs were pretreated with or without RAPA (Rapamycin, 20 nM) for 24h. Then, these cells were harvested and cell lysates were analyzed by Western blot.

**Flow cytometry analysis.**

Liver NPCs were isolated from sham or I/R livers, as described above [38]. A total of 1 x10^6^ cells were incubated with purified rat anti-mouse CD16/32 for 10min and stained with rat anti-mouse F4/80-PE, CD11b-FITC and isotype-matched negative control Abs were added to the cell suspension. After 20min of incubation in the dark, the cells were washed with PBS and subjected to flow cytometric analysis with FACS Calibur (BD Biosciences). For intracellular staining of CD206 and inducible NO synthase, cells were fixed in 4% for maldehyde for 20min after the staining of F4/80 and CD11b, and washed twice with 1x per-meabilization buffer (eBioscience). After incubation with CD206-APC (BioLegend, San Diego, CA) and inducible NO synthase–PE (eBio-science) in 1x per-meabilization buffer for 20min in the dark, the cells were washed with PBS and subjected to flow cytometric analysis.

**Antibodies**

| **Name** | **Description** | **Supplier** | **Cat no.** |
| --- | --- | --- | --- |
| TSC1 | Rabbit | Cell Signaling  Technology | #6935 |
| phos-AKT (Ser473) | Mouse | Cell Signaling  Technology | #23430 |
| AKT | Rabbit | Cell Signaling  Technology | #4691 |
| phos-MST1(T183) | Rabbit | Cell Signaling  Technology | #3681 |
| MST1 | Rabbit | Abcam | ab51134 |
| HMGB1 | Rabbit | Abcam | ab18256 |
| TLR4 | Mouse | Proteintech | 66350-1-Ig |
| NF-kBp65 | Rabbit | Cell Signaling  Technology | #8242 |
| Keap1 | Rabbit | Cell Signaling  Technology | #8047 |
| NRF2 | Rabbit | Cell Signaling  Technology | #12721 |
| Bcl-2 | Rabbit | Cell Signaling  Technology | #3498 |
| Bcl-xl | Rabbit | Cell Signaling  Technology | #2764 |
| β-actin | Rabbit | Cell Signaling  Technology | #4970 |
| GAPDH | Rabbit | Cell Signaling  Technology | #5174 |
| CD11b-FITC | Rat | Abcam | ab24874 |
| F4/80-PE | Rat | Abcam | ab105156 |
| TSC2 | Mouse | Proteintech | 68380-1-Ig |
| Cleaved Caspase 3 | Rabbit | Cell Signaling  Technology | #9664 |
| phos-p70S6K(T389) | Rabbit | Abcam | ab2571 |
| p70S6K | Rabbit | Cell Signaling  Technology | #9202S |
| phos-4E-BP-1(S65) | Rabbit | Cell Signaling  Technology | #9451 |
| 4E-BP-1 | Rabbit | Cell Signaling  Technology | #9452 |
| phos-mTOR(S2448) | Mouse | Proteintech | 67778-1-Ig |
| mTOR | Mouse | Proteintech | 66888-1-Ig |
