# Supplementary material for: Loss of macrophage TSC1 exacerbates sterile inflammatory liver injury through inhibiting the AKT/MST1/NRF2 signaling pathway
Source: Cell Death Dis. 2024 Feb 15;15(2):146. doi: 10.1038/s41419-024-06538-4 (PMC10869801; doi:10.1038/s41419-024-06538-4)
Supplement: Supplementary file 3 — Original Data File [file 41419_2024_6538_MOESM3_ESM.pdf]

Figure 1

Figure 1A

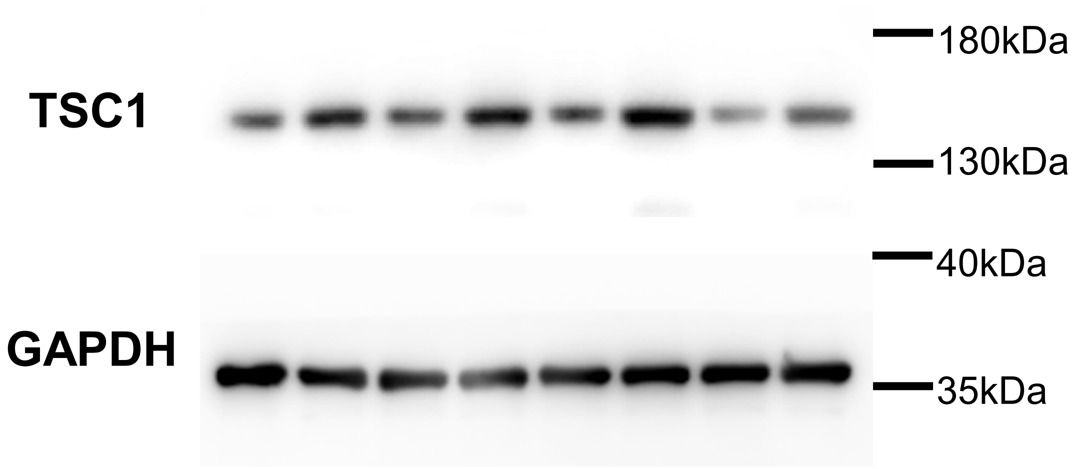

Figure 1B

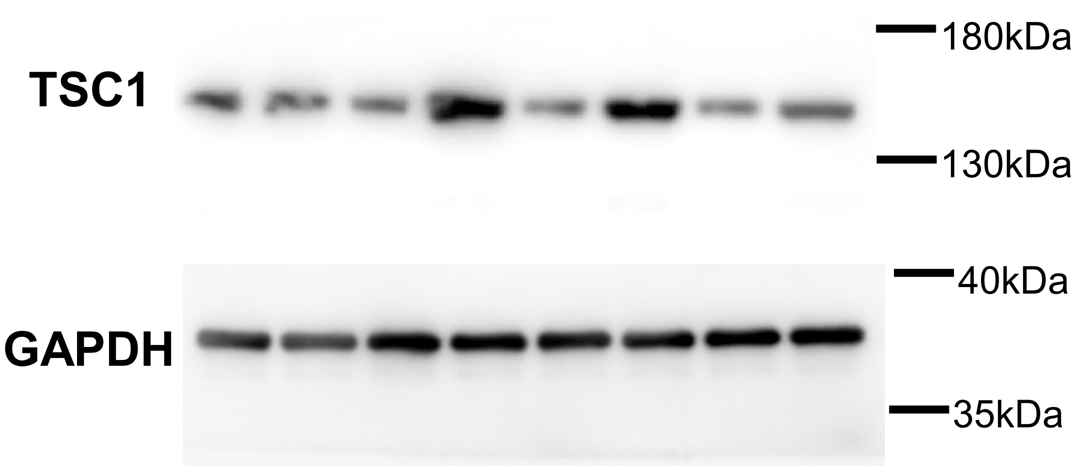

Figure 2

Figure 2A

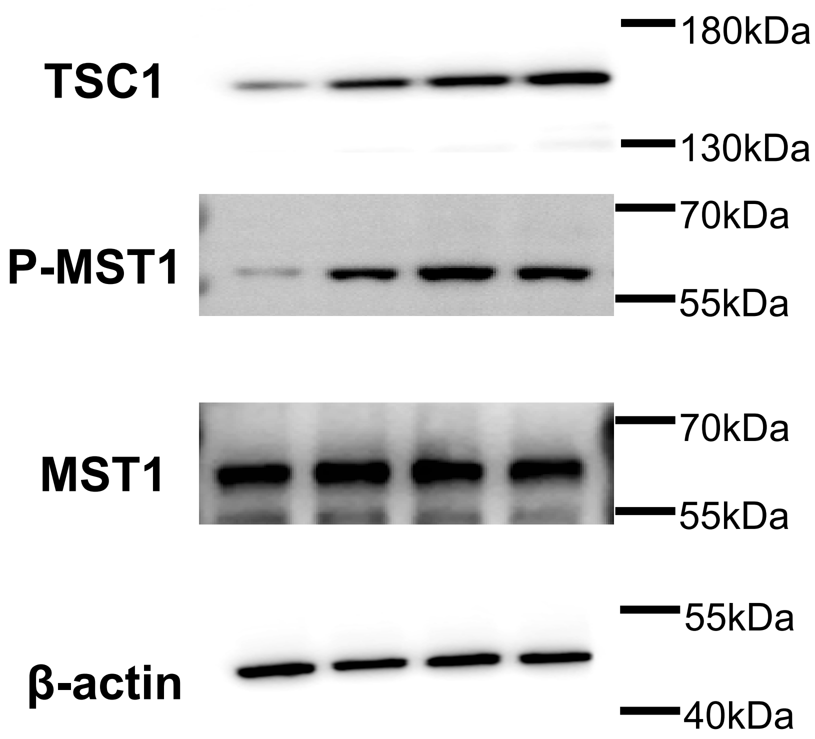

Figure 2B

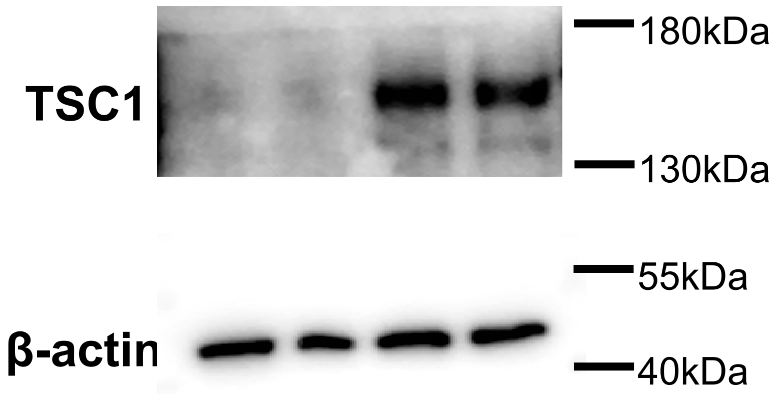

Figure 3

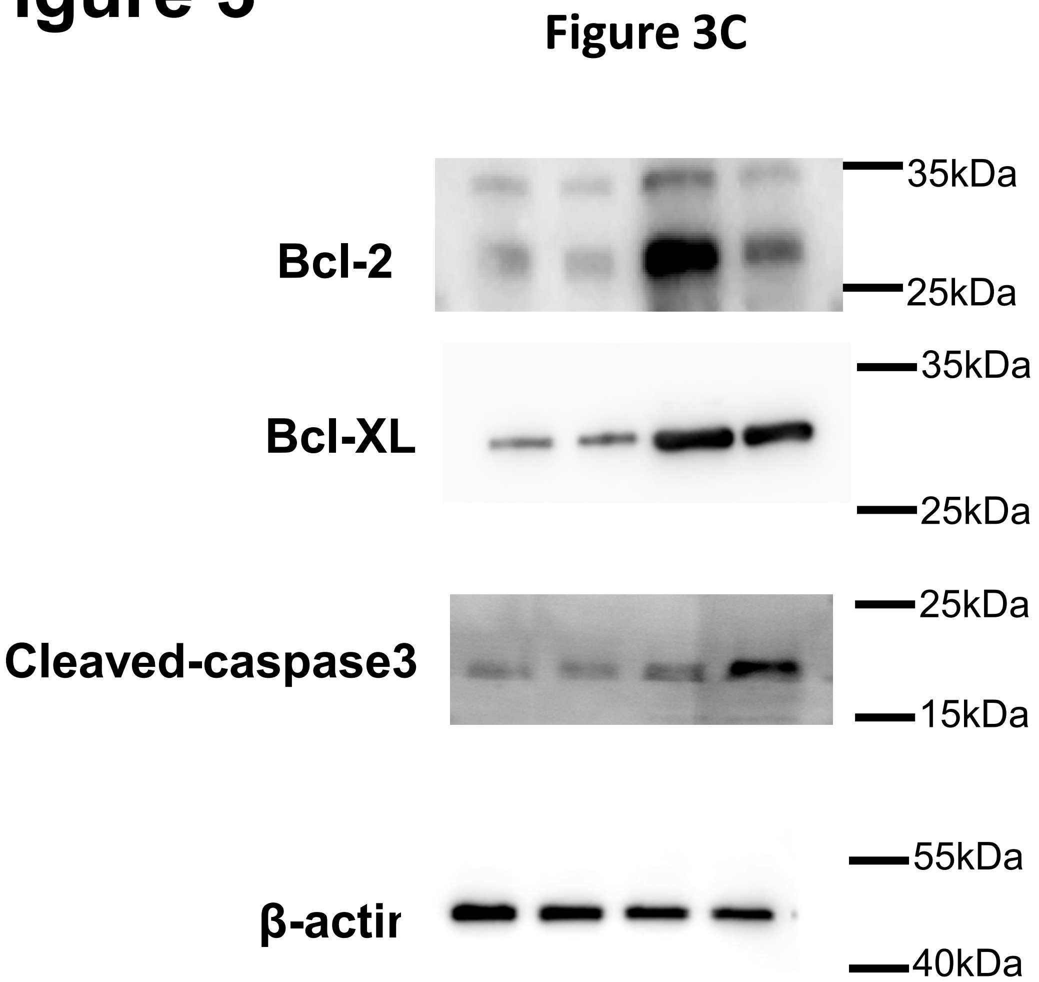
Figure 3C
Bcl-2
35kDa
25kDa
Bcl-XL
35kDa
25kDa
Cleaved-caspase3
25kDa
15kDa
β-actir
55kDa
40kDa

Figure 4

Figure 4C

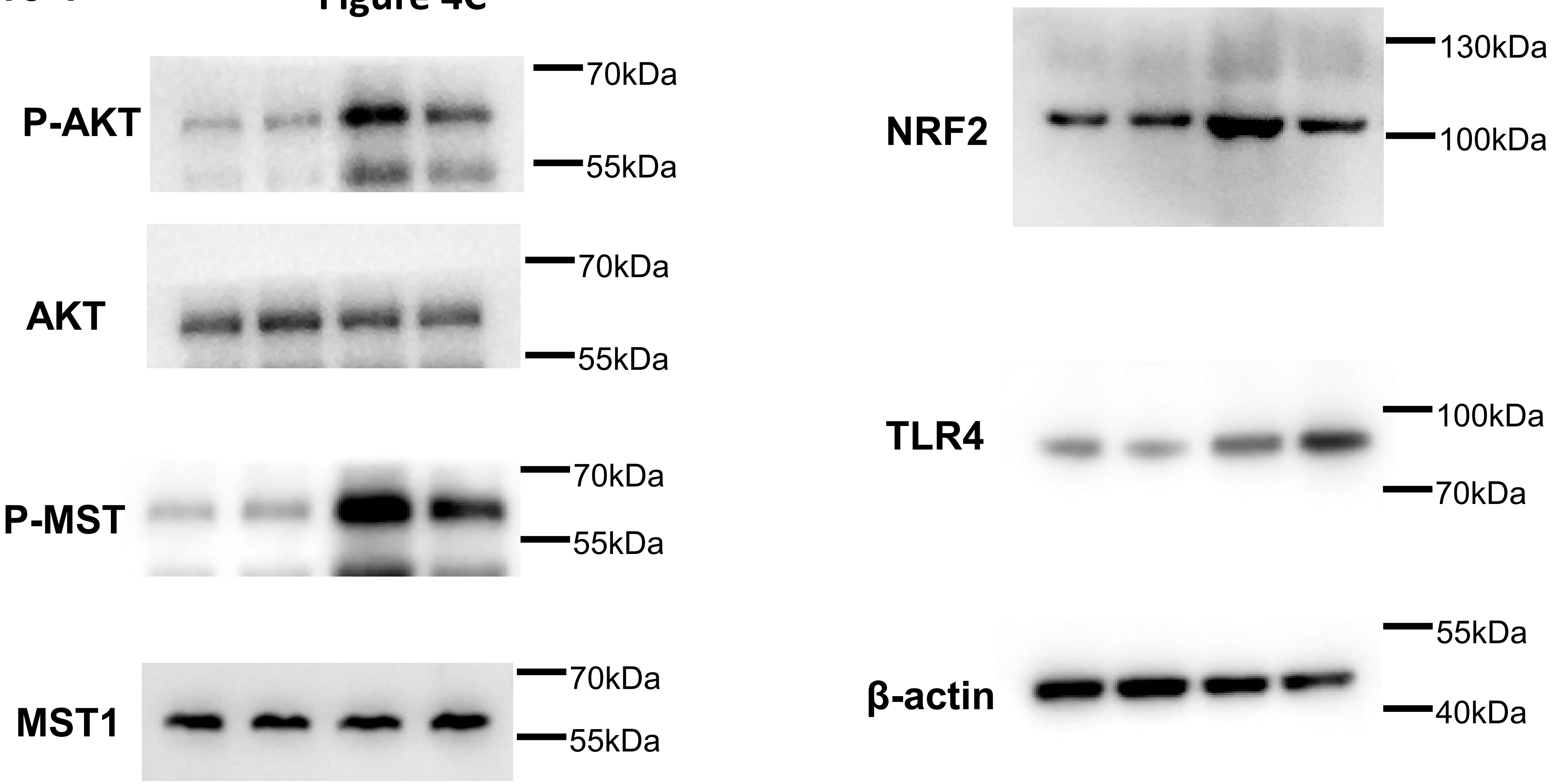

Figure 4

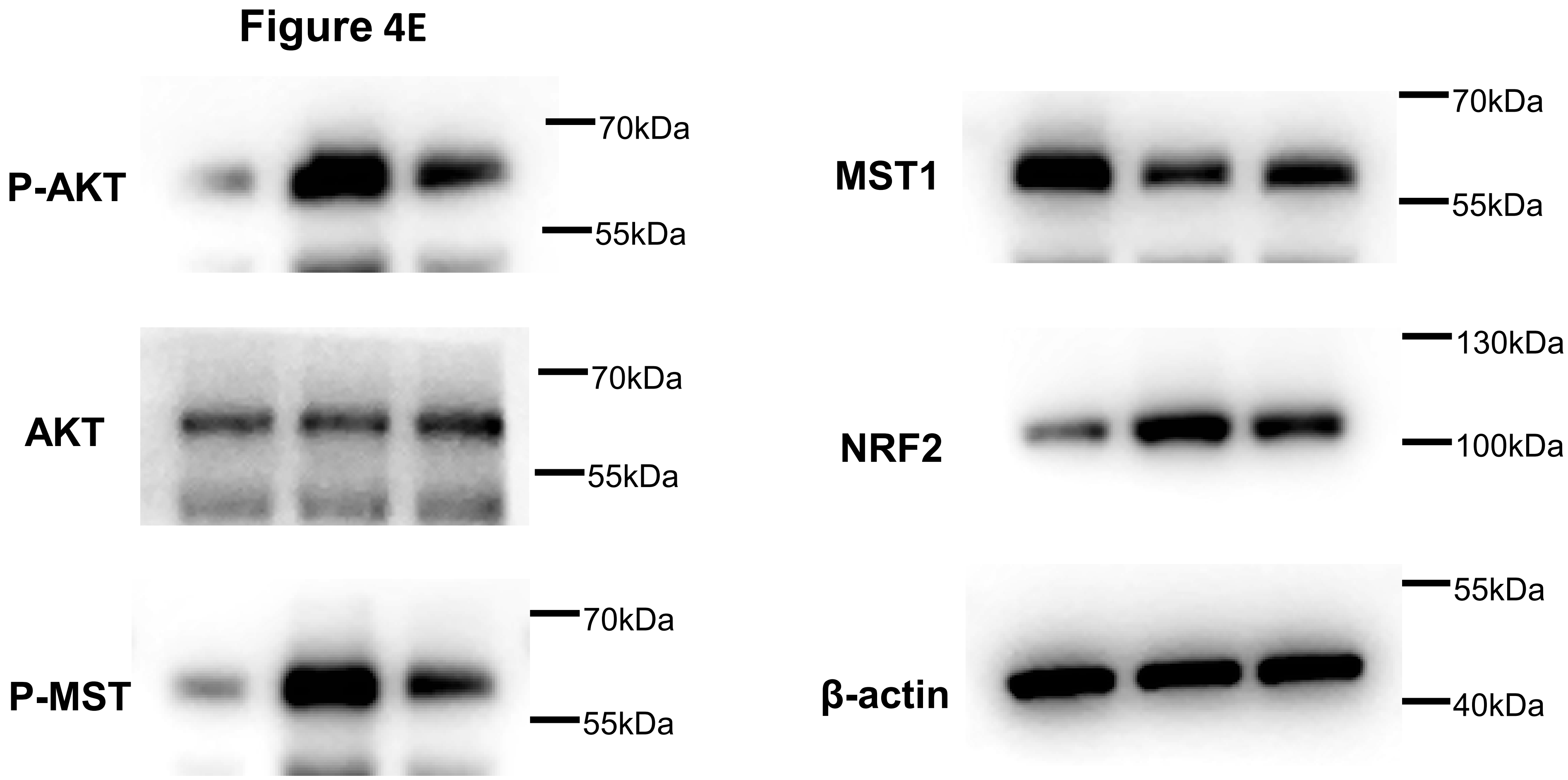
Figure 4E
P-AKT
70kDa
55kDa
AKT
70kDa
55kDa
P-MST
70kDa
55kDa
MST1
70kDa
55kDa
NRF2
130kDa
100kDa
β-actin
55kDa
40kDa

Figure 5

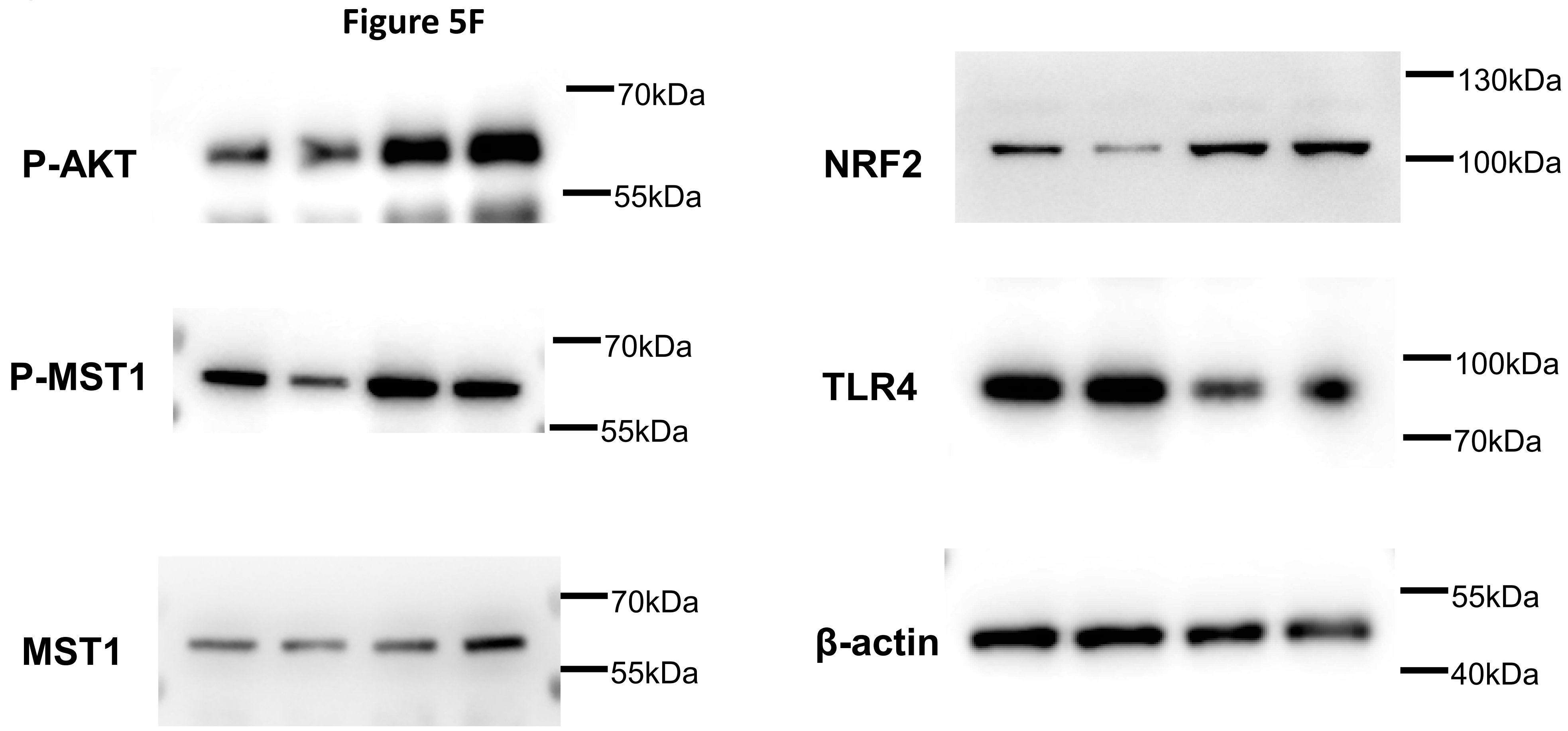
Figure 5F
P-AKT
70kDa
55kDa
P-MST1
70kDa
55kDa
MST1
70kDa
55kDa
NRF2
130kDa
100kDa
TLR4
100kDa
70kDa
β-actin
55kDa
40kDa

Figure 6

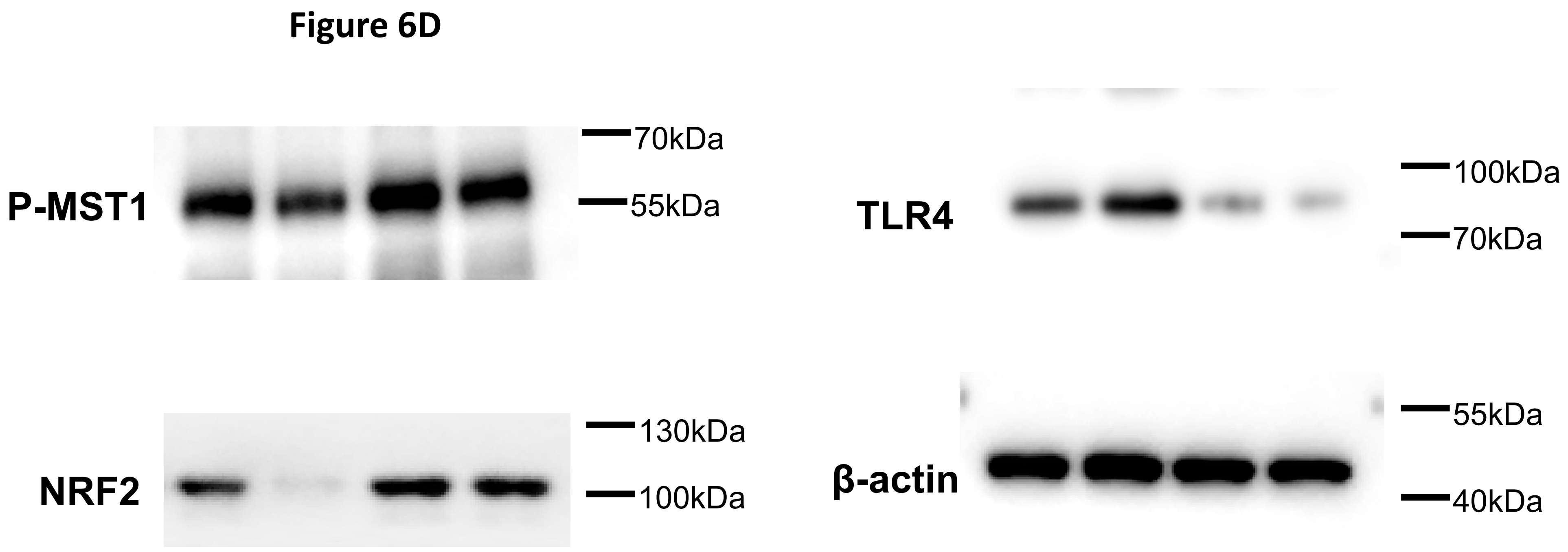
Figure 6D
P-MST1
70kDa
55kDa
NRF2
130kDa
100kDa
TLR4
100kDa
70kDa
β-actin
55kDa
40kDa

Figure 7

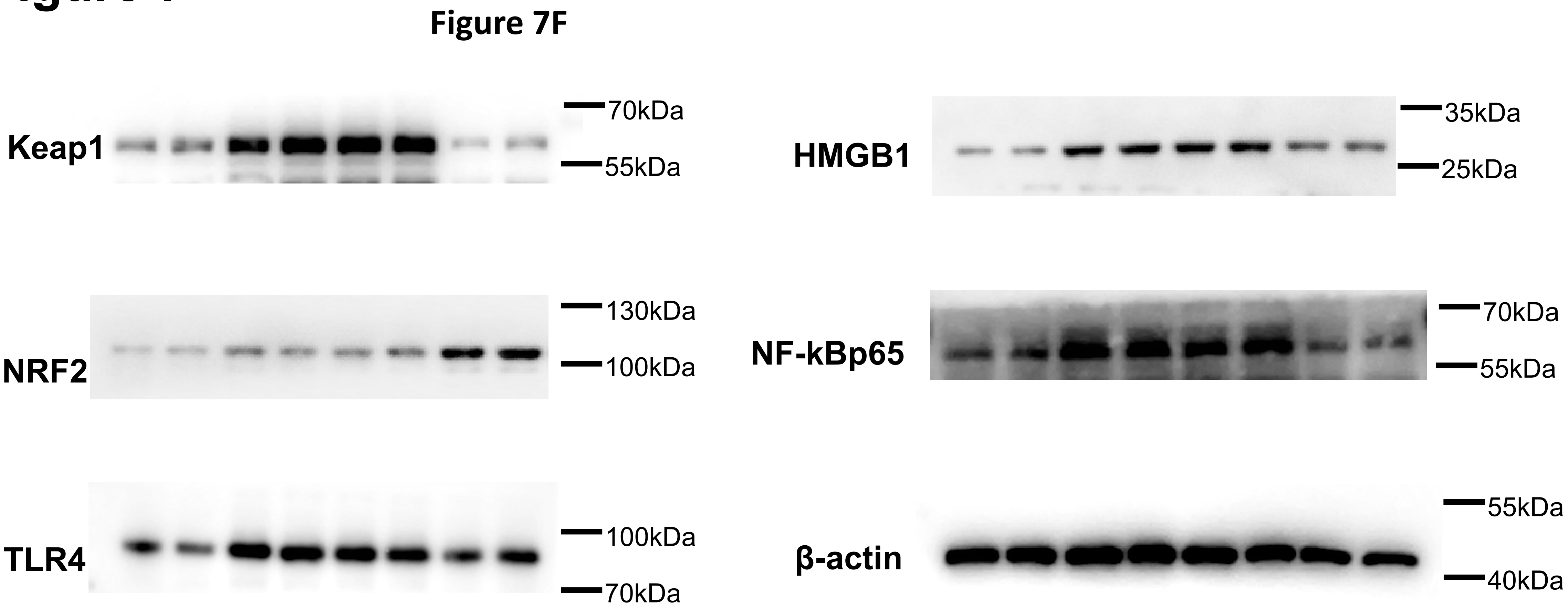
Figure 7F
Keap1
70kDa
55kDa
NRF2
130kDa
100kDa
TLR4
100kDa
70kDa
HMGB1
35kDa
25kDa
NF-kBp65
70kDa
55kDa
β-actin
55kDa
40kDa

Figure 8

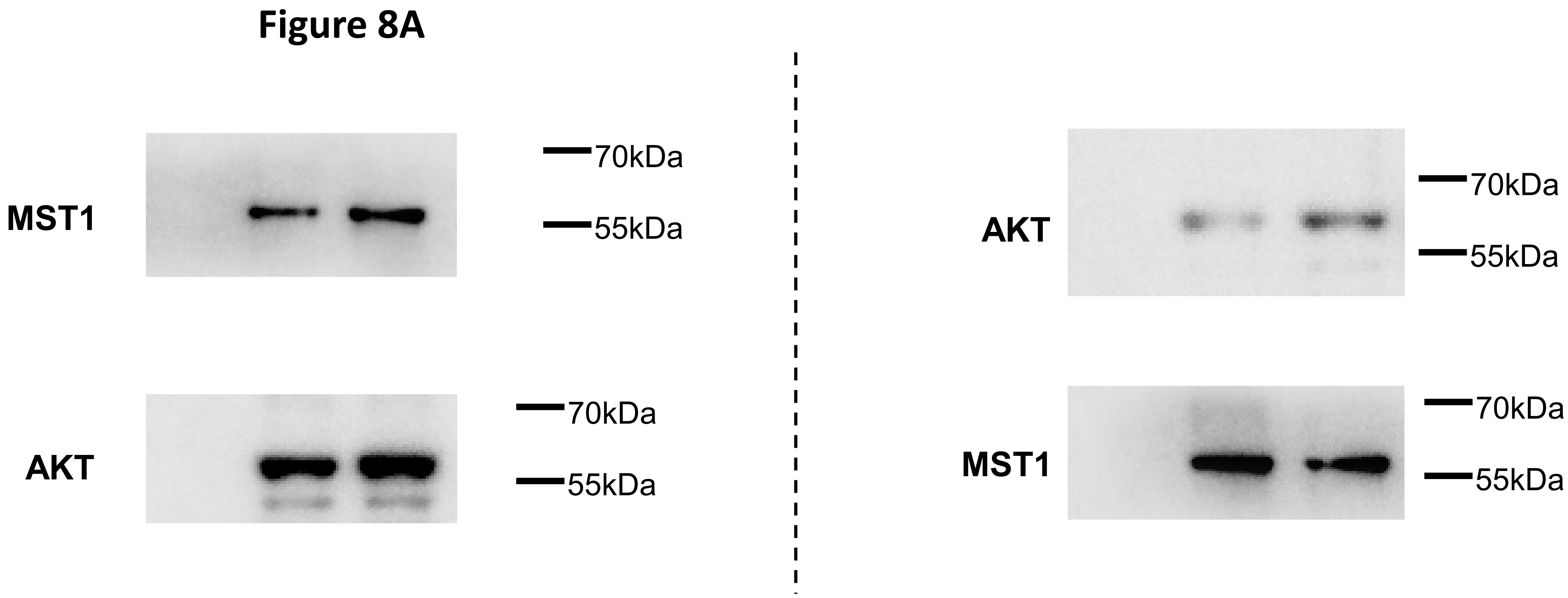
Figure 8A
MST1
70kDa
55kDa
AKT
70kDa
55kDa
AKT
70kDa
55kDa
MST1
70kDa
55kDa

Figure 8

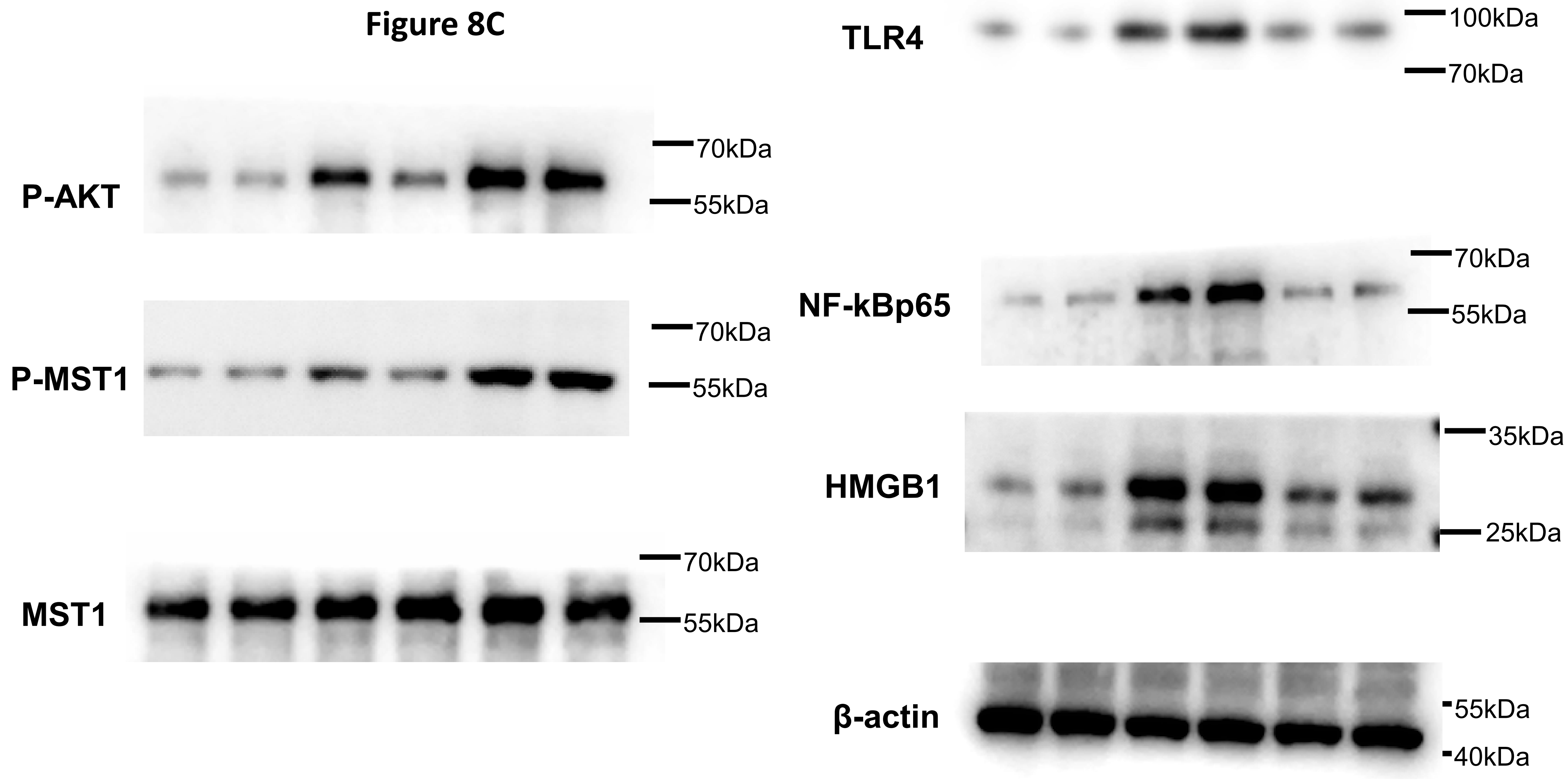
Figure 8C
P-AKT
70kDa
55kDa
P-MST1
70kDa
55kDa
MST1
70kDa
55kDa
TLR4
100kDa
70kDa
NF-kBp65
70kDa
55kDa
HMGB1
35kDa
25kDa
β-actin
55kDa
40kDa

Figure 8

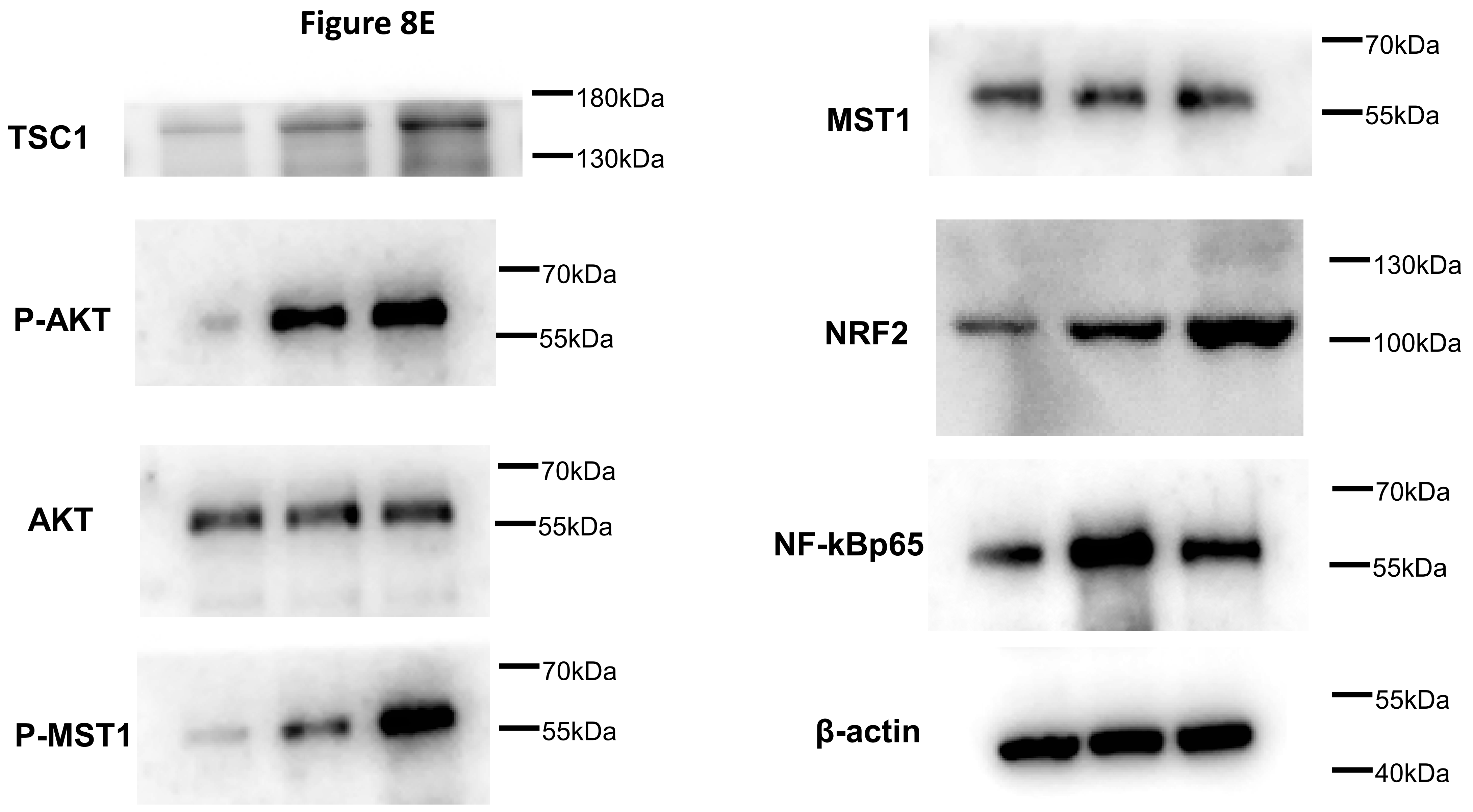

Figure 8E
TSC1
180kDa
130kDa
P-AKT
70kDa
55kDa
AKT
70kDa
55kDa
P-MST1
70kDa
55kDa
MST1
70kDa
55kDa
NRF2
130kDa
100kDa
NF-kBp65
70kDa
55kDa
β-actin
55kDa
40kDa

Figure 9

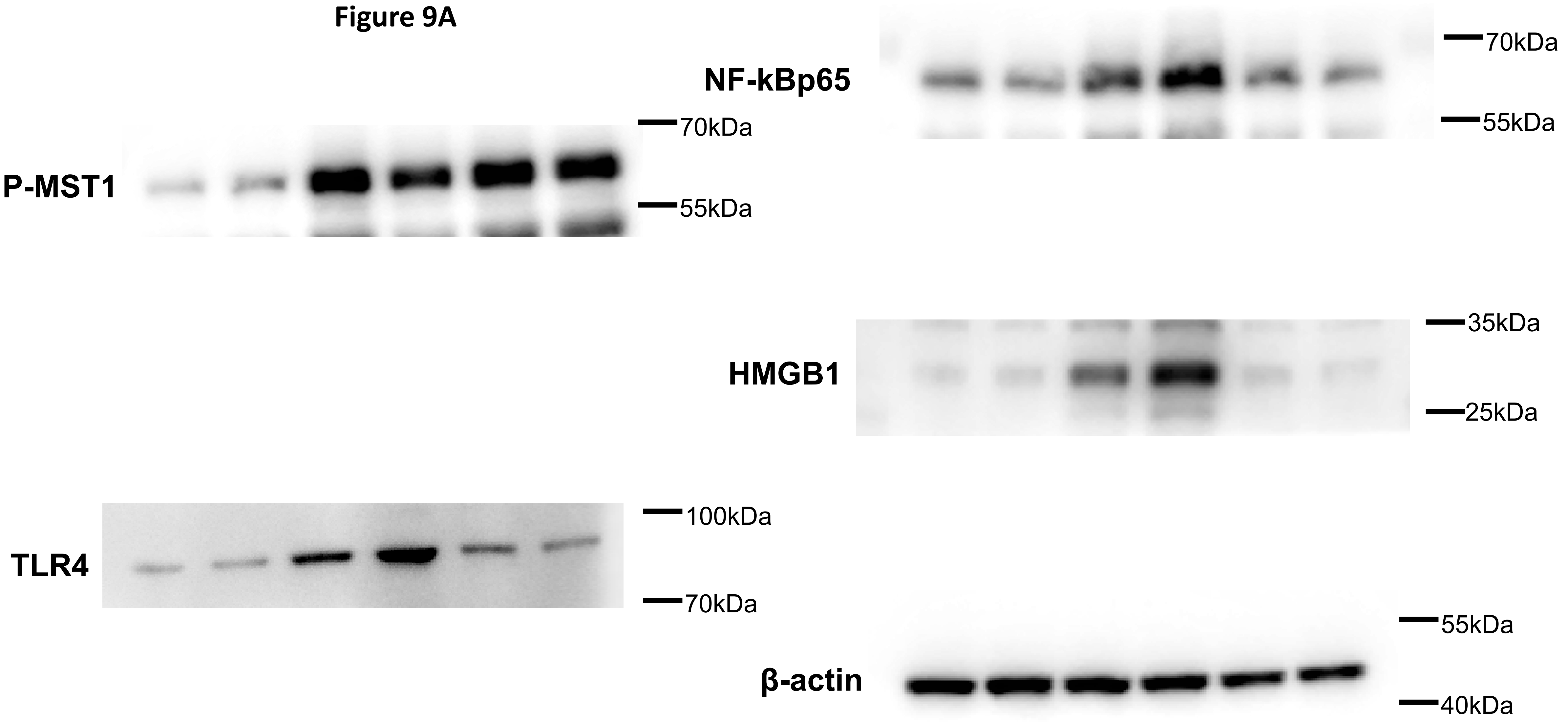
Figure 9A
NF-kBp65
70kDa
55kDa
P-MST1
70kDa
55kDa
HMGB1
35kDa
25kDa
TLR4
100kDa
70kDa
β-actin
55kDa
40kDa

Figure 10

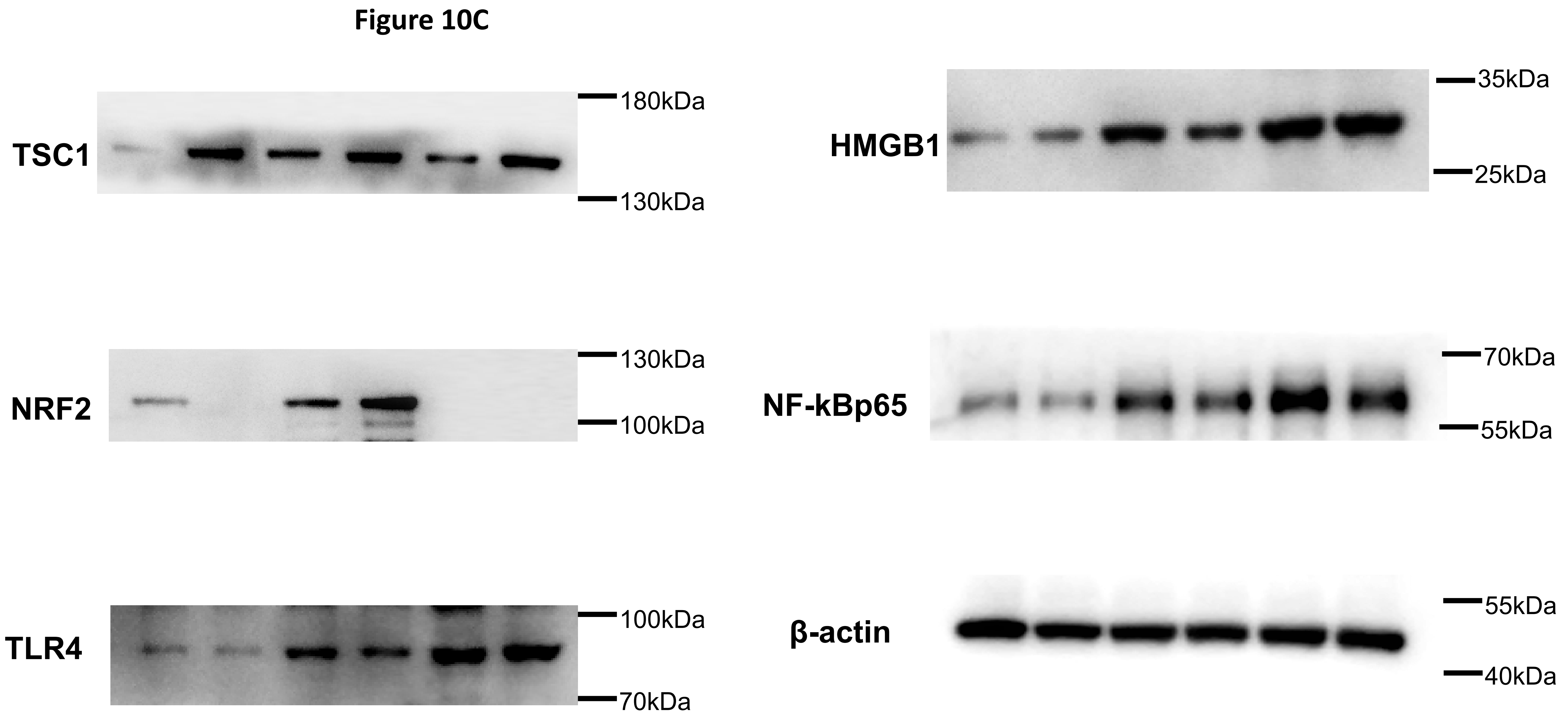
Figure 10C
TSC1
180kDa
130kDa
NRF2
130kDa
100kDa
TLR4
100kDa
70kDa
HMGB1
35kDa
25kDa
NF-kBp65
70kDa
55kDa
β-actin
55kDa
40kDa

Figure 10

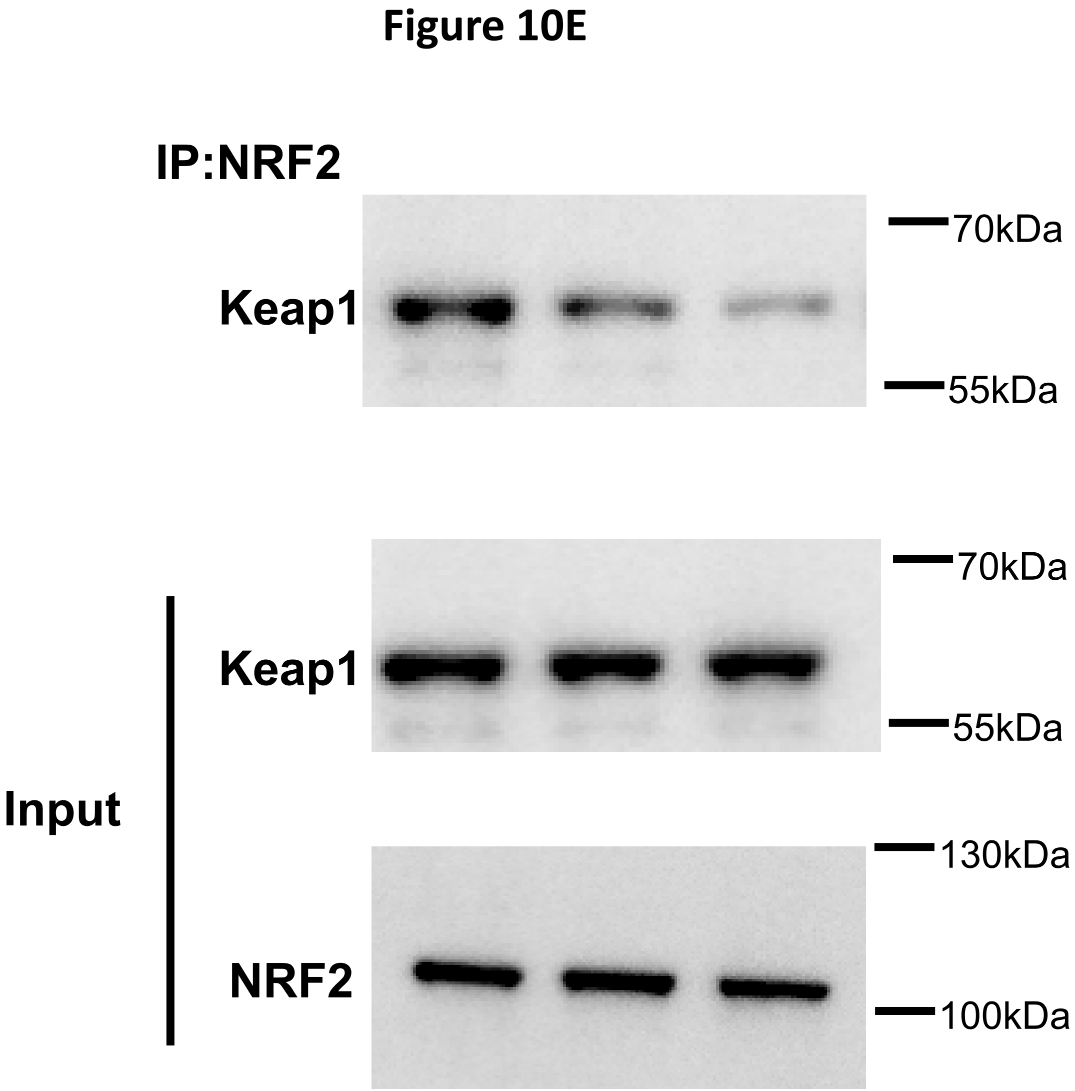
Figure 10E
IP:NRF2
Keap1
70kDa
55kDa
Keap1
70kDa
55kDa
Input
NRF2
130kDa
100kDa

Figure 10

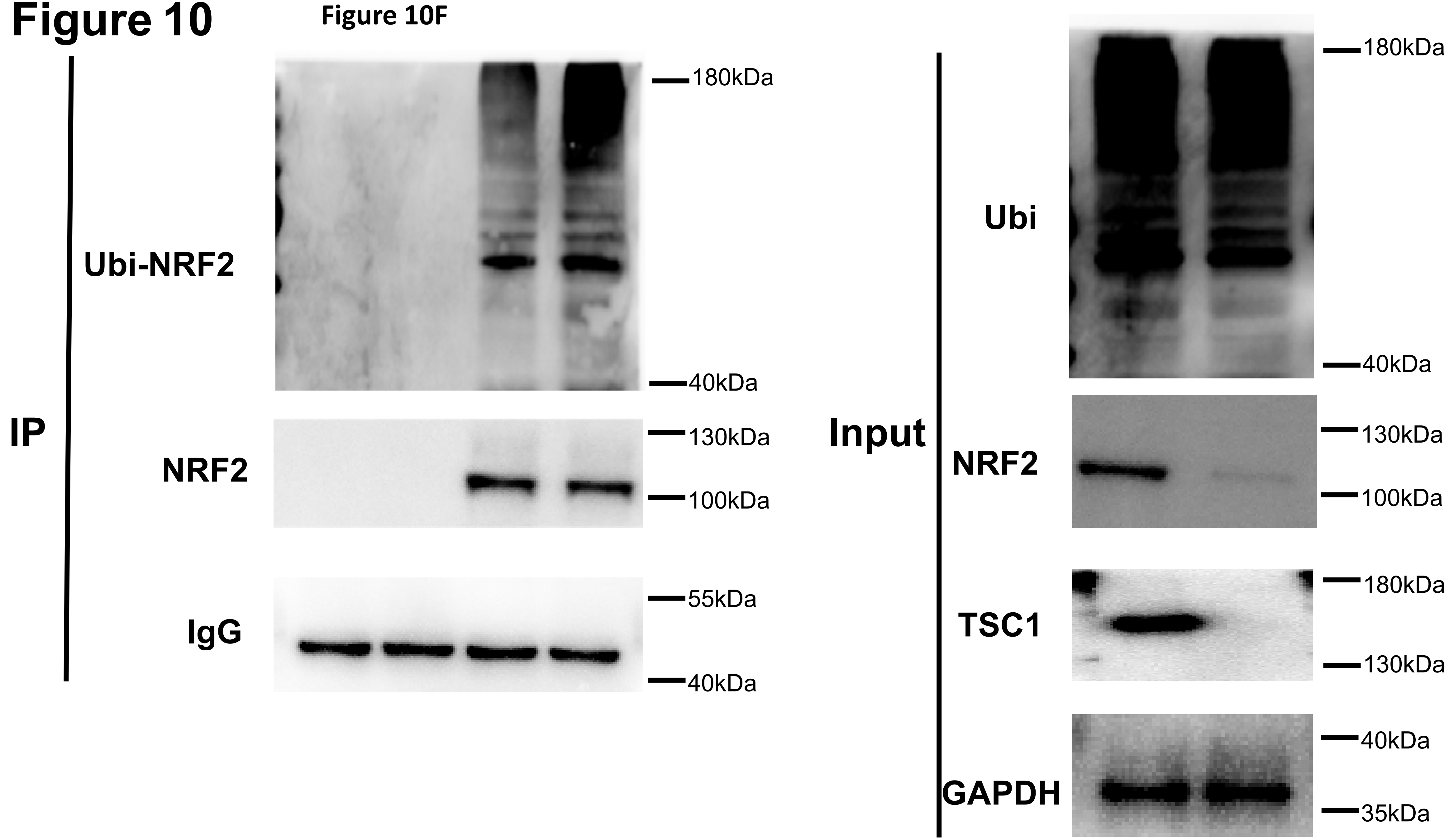
Figure 10F
IP
Ubi-NRF2
180kDa
40kDa
NRF2
130kDa
100kDa
IgG
55kDa
40kDa
Input
Ubi
180kDa
40kDa
NRF2
130kDa
100kDa
TSC1
180kDa
130kDa
GAPDH
40kDa
35kDa

Figure S1

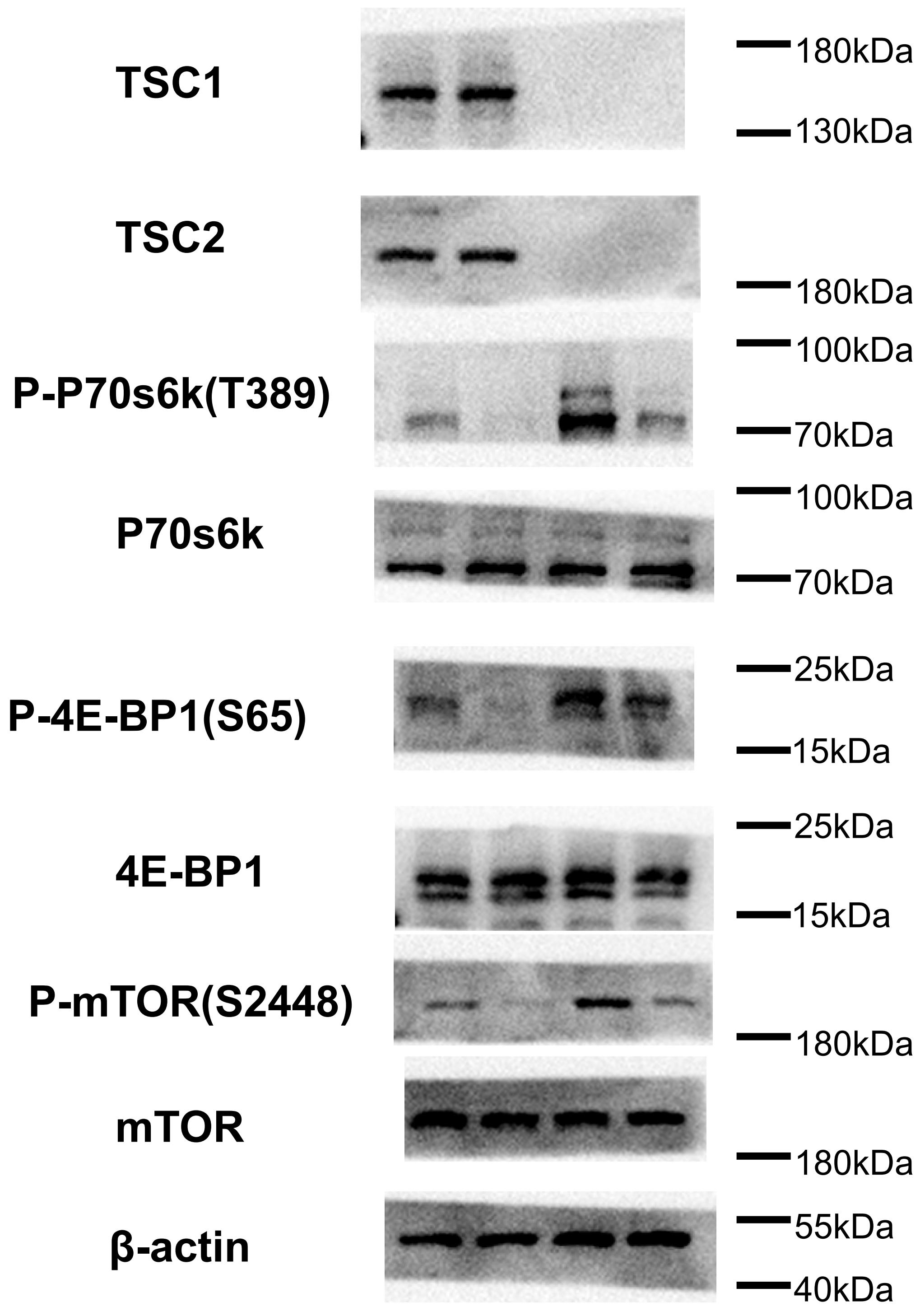

TSC1
180kDa
130kDa
TSC2
180kDa
P-P70s6k(T389)
100kDa
70kDa
P70s6k
100kDa
70kDa
P-4E-BP1(S65)
25kDa
15kDa
4E-BP1
25kDa
15kDa
P-mTOR(S2448)
180kDa
mTOR
180kDa
β-actin
55kDa
40kDa
